# Nonideal State Equations to Evaluate the Laminar Flame Speed and Ignition Delay Times at Subcritical, Transcritical, and Supercritical Conditions of Ethanol

**DOI:** 10.1021/acsomega.4c09415

**Published:** 2025-05-30

**Authors:** Paulo Vitor Ribeiro Plácido, Henrique Beneduzzi Mantovani, Dario Alviso, Rogério Gonçalves dos Santos

**Affiliations:** † School of Mechanical Engineering, 28132 University of Campinas (UNICAMP), Rua Mendeleyev, 200 - CEP 13083-860, Cidade Universitária “Zeferino Vaz”, Barão Geraldo, Campinas, SP 13086-002, Brazil; ‡ Laboratorio de Mecánica y Energía, Facultad de Ingeniería, Universidad Nacional de Asunción, Campus Universitario, CP 2160 San Lorenzo, Paraguay; § Laboratorio de Fluidodinámica, Facultad de Ingeniería, Universidad de Buenos Aires/CONICET, Paseo Colón 850, CP 1063 Buenos Aires, Argentina

## Abstract

Several studies have been conducted to identify an efficient method for reducing particulate emissions from vehicle exhaust gases, which are significant contributors to air pollution in large urban areas. One promising approach involves using supercritical combustion, injecting fuel directly at its critical temperature and pressure. Supercritical fluids possess a lower viscosity and surface tension than liquids and higher diffusion rates, facilitating a more uniform mixture distribution, enhancing thermal efficiency, and reducing particulate emissions. This study focuses on investigating ethanol supercritical combustion as a viable biofuel option. It proposes a simplified kinetic mechanism comprising 53 species and 385 reactions derived through sensitivity analysis and directed relation graph error propagation. To validate this mechanism, simulations were conducted using Cantera with a cubic Peng–Robinson (PR), Redlich–Kwong (RK), and an ideal (I) equation of state (EoS) for 1D laminar flame speed (LFS) and 0D constant-volume autoignition delay time (IDT) simulations for anhydrous ethanol. The IDT results agreed with experimental data across a temperature range of 700–1250 K at 10, 30, 50, 75, and 80 atm, showing good agreement with LFS experiments conducted at 298–949 K, 1–10 atm, and (ϕ) of 0.6–1.8. A normalized computational time ratio was calculated for each EoS relative to the ideal gas, revealing computational costs almost seven times higher for R–K and nearly nine times higher for P–R EoS compared to the ideal gas EoS. The study also examined the limitations of the ideal gas equation of state (EoS) in capturing real gas effects, particularly under ultrahigh-pressure conditions (greater than 100 atm), which revealed significant disparities in simulations at 500 atm. The results indicate that while the ideal gas EoS suffices for ethanol under atmospheric and transcritical conditions, a real gas EoS is crucial for accurate simulations under ultrahigh-pressure conditions.

## Graphical abstract

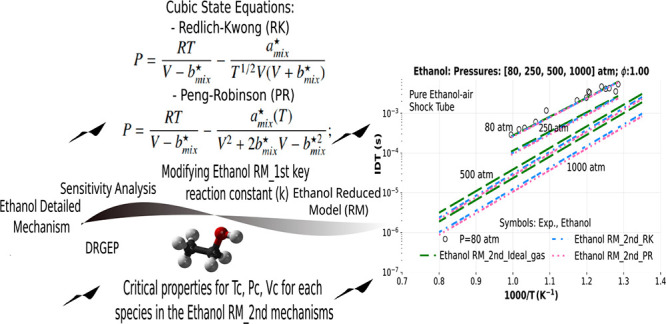

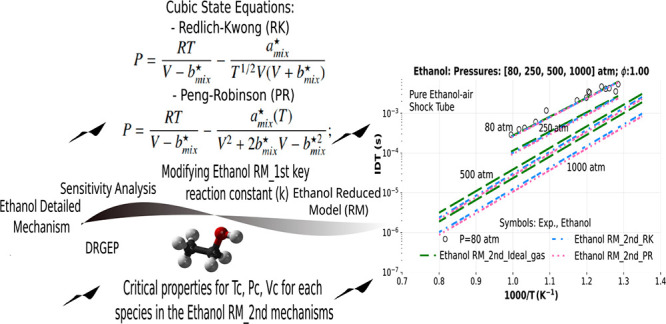

## 1 Introduction

In recent history, the expansion of vehicle transportation and the use of fossil fuels have increased rapidly due to societal advancements, particularly in major cities. The global focus has been directed toward reducing the impacts of vehicle emissions and air pollution on climate change and public health. Consequently, there is a demand for clean and highly effective combustion systems. Given these challenges and the possibility to improve the thermal efficiency of combustion, a method known as a supercritical environment for fuel has been proposed to address these concerns and create a high-temperature and high-pressure setting in the combustion system during operation conditions.^1,2^ This method, widely recognized and successfully employed in liquid rockets, diesel, and aircraft engines,^3^ has recently been examined in the work of Schmitt,^4^ where they conducted large-eddy simulations on the Mascotte test cases under supercritical pressure.

Due to their different properties, these supercritical fluids (SCFs) are an attractive medium for chemical reactions. According to Liu et al.,^5^ supercritical fluids have lower viscosity and surface tension than liquids; on the other hand, they have higher diffusion rates,^4^ causing an effectively distribution of fuel and air, enhancing thermal efficiency and decrease particulate emissions from the combustion systems when it is injected into a reactors, such as cylinders, in a supercritical environment.^2^

Using biofuels, such as ethanol, as a standalone fuel or mixed with regular gasoline, like E85 (85% ethanol and 15% gasoline), is another way to control emissions. In Brazil, it is common to find flexible fuel combustion engine systems that can operate using several gasoline and ethanol mixtures.^6^ Ethanol brings several other benefits, such as renewability, lower carbon and pollutant emissions, and engine knock control,^7^ and it can be a renewable building block for fuels and chemicals.^8^ Additionally, there is a push to reduce carbon emissions and other pollutants from traditional energy and power sources (hydrocarbon fuels, such as gasoline and diesel). These needs are rooted in a desire to be more economically and environmentally conscious.

In recent years, research has been focused on enhancing the performance of gasoline and flex-fuel combustion systems by developing and applying high and ultrahigh-pressure injection (UHPDI) technology to optimizing thermal efficiency while reducing particle emissions, once it depends on the quality of fuel mixture/air, which is closely linked to fuel breakup, diffusion, atomization, and vaporization of the fuel injecting conditions generating a homogeneous mixture condition, as claimed by Yamaguchi et al.^9,10^ and Li et al.^11,12^

Their analyses at a range of 50–150 MPa reveal that increased injection pressure enhances the vortex scale, thereby improving air/fuel mixing quality. They also identify UHPDI as a potential method to improve air/fuel mixture homogeneity for combustion systems fueled with ethanol or gasoline/ethanol blends.

For instance, studying experimental ignition delay times and laminar flame speeds is essential for validating the chemical kinetic models used in high-pressure combustion simulations. These studies must consider real gas behavior in both chemical kinetics and thermodynamic properties, particularly because significant species can reach transcritical states. In a transcritical state, only temperature or pressure exceeds the critical values for specific fuel species, and supercritical states occur when both temperature and pressure surpass it.^13,14^

However, experimental data often lack coverage of the supercritical region, while chemical kinetics models typically assume an ideal gas state equation for reacting mixtures at elevated pressures, such as in Roy and Askari^15^ for ethanol, Zhang et al.^16^ examining the spray features of a diesel surrogate fuel composed of six components at various injection pressures, and Harman-Thomas et al.^17^ conducted research on the combustion of carbon dioxide at supercritical conditions.

Moreover, only a few studies have incorporated real gas equations of state (EoS).^18–20^ There is limited research on long-chain hydrocarbon fuels that considers the effects of real gas. For example, Kogekar et al.^21^ examined the real gas effects using a multicomponent Redlich–Kwong (R–K) equation by comparing experiments of high-pressure shock tube (ST) ignition delay time (IDT) data and combustion simulations concerning mixtures of n-dodecane/O_2_/N_2_. The kinetics model used in their study was provided by Wang et al.,^22^ comprising 100 species and 432 reactions. Their results indicated that real gas behavior impacted the simulated IDTs in the negative temperature coefficient (NTC) region by 50–100 μs.

As reference values, critical properties, such as critical temperature and pressure, are shown in Table 1 for alcohol fuel (ethanol), oxygen, nitrogen, and argon.

**Table 1:** Critical Properties of Selected Species Fluids from Poling et al.,^13^ and Yaws^23^

formula	name	*T*_c_ (K)	*P*_c_ (bar)
C_2_H_6_O (C_2_H_5_OH)	ethanol	513.9	61.48
O_2_	oxygen	154.6	50.43
N_2_	nitrogen	126.2	33.98
Ar	argon	150.86	48.98

Several kinetic models describe the reaction mechanism of ethanol oxidation using ideal gas equations of state (EoS). Marinov’s mechanism^24^ was one of the earliest detailed models of ethanol oxidation. More recent kinetic mechanisms have also been published. These models, such as those found in Cancino et al.,^25^ Lee et al.,^26^ Konnov et al.,^27^ Metcalfe et al.,^28^ Cai and Pitsch,^29^ Roy and Askari,^15^ Marques and da Silva,^30^ Shi et al.,^31^ and Jin et al.^32^ have significantly improved the description of ethanol combustion chemistry concerning numerical validations against experimental high-pressure shock tubes, rapid-compression machines, jet stirred reactors, and counter-flow diffusion flames data, such as ignition delay times, species profile data, and laminar flame velocity measurements. However, no study in the literature is available regarding transcritical and supercritical anhydrous ethanol combustion using the real gas equation of state in both laminar flame speed (LFS) and ignition delay time (IDT).

Therefore, this work focuses on developing a new reduced mechanism to predict the oxidation of transcritical and supercritical ethanol. This model uses the real gas cubic Peng–Robinson (P–R) and Redlich–Kwong (R–K) equations of state (EoS) to account for nonideal effects on ethanol laminar flame speed (LFS) and ignition delay time (IDT) simulations. The final ethanol kinetic mechanism consists of 53 species and 385 reactions. The simulations involve a 0D constant-volume IDT and 1D LFS. The model is validated against experimental results under high-pressure conditions of IDT at shock tube (ST), stoichiometric equivalence ratio (ϕ), high-pressure range (10–80 atm), and temperatures between 700 and 1250 K. Additionally, the study includes LFS validations at 1–10 atm and 298–949 K using the ideal EoS, P–R EoS, and R–K EoS implemented in Cantera version 3.0.0, with the Helmholtz free energy definition. The critical properties of each species in the model are obtained to determine the intermolecular interaction parameters (*a* ^★^) and (*b* ^★^) used in the cubic R–K and P–R equations of state and Joback’s Group Method. The impact of using a real gas state equation on the LFS and IDT computational costs is also analyzed.

## 2 Kinetics Modeling

### 2.1 Selection of the Ethanol Original Base Model

To develop a new reduced ethanol chemical kinetics mechanism and conduct 1D and 0D combustion numerical simulations using real gas state equations, and to avoid starting from scratch, many chemical kinetics mechanisms for ethanol combustion can be found in the literature^15,24,25,28,30,31,33–35^ and can be used as a base mechanism for the reduction process. These mechanisms, whose development and validations occur at atmospheric, intermediate, or high-pressure conditions, have typically used ideal gas equations of state for validations.

The selection of the base mechanism for utilizing real gas equations should be based on its ability to accurately agree with the high-pressure conditions of interest. Moreover, the original mechanism’s complexity (number of species and reactions), which affects computational costs and the new parameters introduced in the real gas state equation due to molecular interactions (such as the repulsive correction *b* ^★^ and the attraction parameter *a* ^★^ in the Redlich–Kwong EoS^21^), also needs to be considered. The challenge lies in the fact that these parameters must be determined by the critical properties of each species in the chosen mechanism. However, critical values are typically unavailable for radical and intermediate species, as discussed later in the real equations of State section.

Therefore, four recent and interesting high-pressure ethanol chemical kinetic mechanisms from the literature were analyzed and are presented in Table 2, considering the validation range of each model.

**Table 2:** High-Pressure Chemical Kinetics Mechanisms Comprising Ethanol (E) Available in the Literature^a^

reactions	species	*P* (atm)	*T* (K)	ER ϕ	validation	ref
3037	581	0.65–75	298 – 2500	0.4–2.0	FR, IDT, JST, LFS, FSP, RCM,	(AramcoMech 3.0)^28^
1795	107	1.0–30	298–1450	0.5–2.0	IDT, LFS, RCM, ST	Zyada et al.^35^
1016	67	1.0–50	300–1450	0.3–2.0	IDT, LFS, JSR, FSP, ST, RCM	Roy et al.^15^
188	43	1.0–50	358–1430	0.3–1.0	IDT, LFS, ST, RCM	Marques et al.^30^

a ER ϕ, Equivalence ratio; IDT, ignition delay times; JSR, jet stirred reactors; LFS, laminar flame speed; RCM, rapid compression machine; FSP, flame species profile; ST, shock tube.

To emphasize the most appropriate agreement of the models at high-pressure shock tube conditions and support the selection of the base mechanism from Table 2, Figure 1 compares the numerical ignition delay time (IDT) with shock tube experimental data from Cancino et al.^25^ at 50 atm and that from Lee et al.^26^ at 80 atm. This range of pressure and temperature is notably valuable for featuring transcritical and supercritical conditions for the ethanol fuel critical properties species (see Table 1).

**Figure 1:**
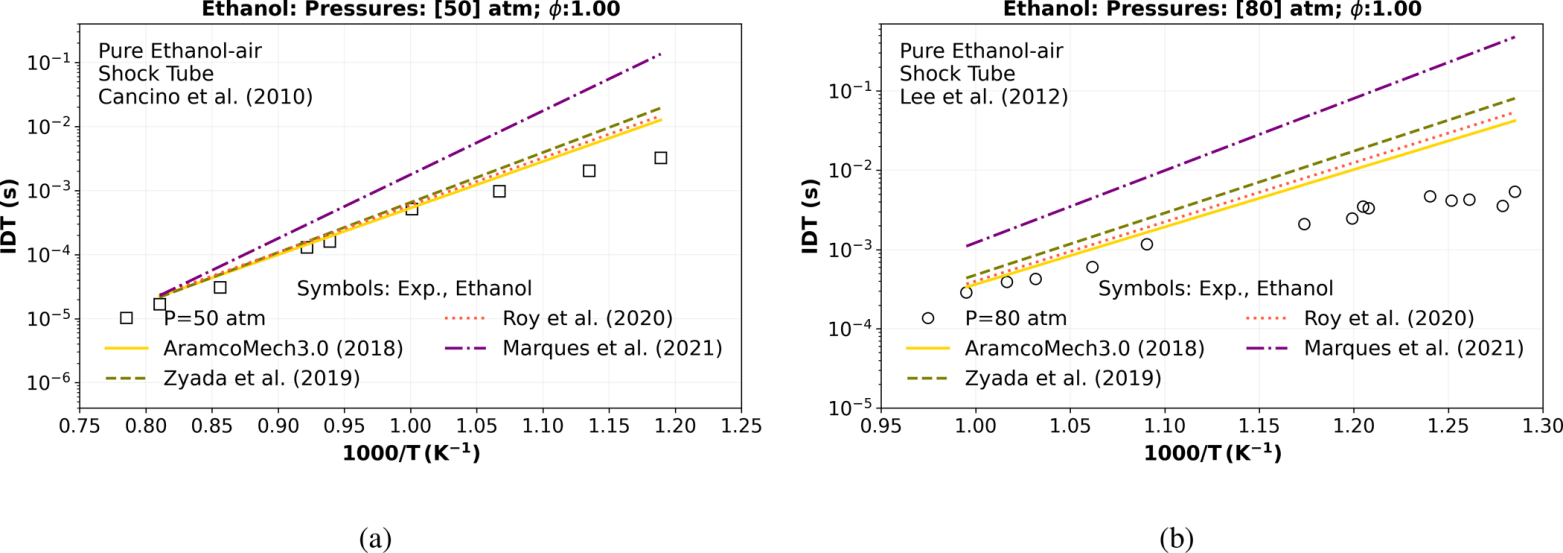
Numerical stoichiometric (ϕ = 1) IDT simulation at different pressures considering all the ethanol mechanisms^15,28,30,35^ in Table 2 versus the experimental high-pressure shock tube data of (a) Cancino et al.^25^ at 50 atm (square) and (b) of Lee et al.^26^ at 80 atm (circle).

Concerning Figure 1a,b, the best agreement with both shock tube experimental data at 50 atm (square)^25^ and 80 atm (circle)^26^ is obtained by the original (C1–C4) mechanism of Metcalfe et al.^28^ (−), known as AramcoMech3.0 (2018) from the Galway database, which has been widely validated considering ethanol data in the literature. Unfortunately, this mechanism presents many species and reactions, and consequently, the computational costs are very high when it is used in 1D LFS simulations.

The second-best agreement is from Roy and Askari^15^ (···), a semidetailed mechanism of ethanol developed in 2020 using a reaction mechanism generator (RMG) to predict the performance of this fuel in engine-relevant operating conditions.

Considering the ignition delay time verification, it presents a good agreement at lower and intermediate pressures (≤30). However, unfortunately, their numerical results of IDT over 30 atm present deviations from the high-pressure IDT shock tube experienced at temperatures below 950 K, as observed in Figure 1a,b.

The third best agreement is from Zyada and Samimi-Abianeh^35^ (− – −), a detailed kinetic mechanism for ethanol generation using RMG in 2019. Their study successfully applied the heat transfer effect of the RCM calculation in a numerical simulation. However, it is important to note that this new mechanism has some limitations in IDT at high-pressure calculations, tested between 1 and 30 atm in their study, which is the lowest pressure range of all mechanisms summarized in Table 2.

The highest deviation observed in Figure 1a,b is from the Marques et al.^30^ (− · −), a reduced mechanism for ethanol under ultralean engine conditions published in 2021. This mechanism consists of 43 species and 188 reactions. Although their simulation results agreed with ignition delay times experimental data at various pressures, especially at 30 bar (ϕ = 0.3),^25^ and at 40 bar (ϕ = 0.5),^36^ when considering stoichiometric and rich conditions (ϕ ≥ 1), the numerical results deviate far from the ignition delay times experimental data, as well observed in Figure 1.

Considering all the above information, the ethanol model by Metcalfe et al.^28^ (AramcoMech3.0, 2013–2018) was chosen as the base mechanism for ethanol. Additionally, two reduction techniques were employed to manage the high number of species and reactions in the ethanol-based model.

### 2.2 Reduction Process

This work achieved the ethanol-reduced model by reducing the AramcoMech3.0 model^28^ (581 species and 3037 reactions). The reduction approach is a combination of the sensitivity analysis (SA)^37^ and the Directed relation graph with error propagation (DRGEP).^38^ All these features are available in Pymars code,^39^ version 1.1.0.

A sensitivity analysis of a stoichiometric IDT ethanol/air mixture based on the concentration of [OH] was conducted to identify species associated with key reactions under high-pressure conditions (50 atm), as graphically represented in Figure 2a. These identified species constitute the primary ethanol oxidation route species under high pressure, whose oxidation path is dominated by hydrogen-atom abstraction by the hydroperoxy radical (HO_2_), producing CH_3_CHOH, as demonstrated in Figure 2b, and previously observed in the ethanol study of Cancino et al.^25^ These key reactions were designated as target species in the reduction process. Consequently, the targeted species within the DRGEP encompassed OH, CO, the hydroperoxy radical (HO_2_), acetaldehyde (CH_3_CHO=C_2_H_4_O), hydrogen peroxide (H_2_O_2_), and 1-hydroxyethyl radical CH_3_CHOH (referred to as “SC2H4OH” in,^28^ one of the three isomers of C_2_H_5_O), due to their pronounced sensitivity to pressure, as depicted in Figure 2a. The selected target combustion products were CO_2_ and H_2_O. These analyses guarantee that the high-pressure ethanol oxidation route identified in the original base mechanism is preserved through the reduction process.

**Figure 2:**
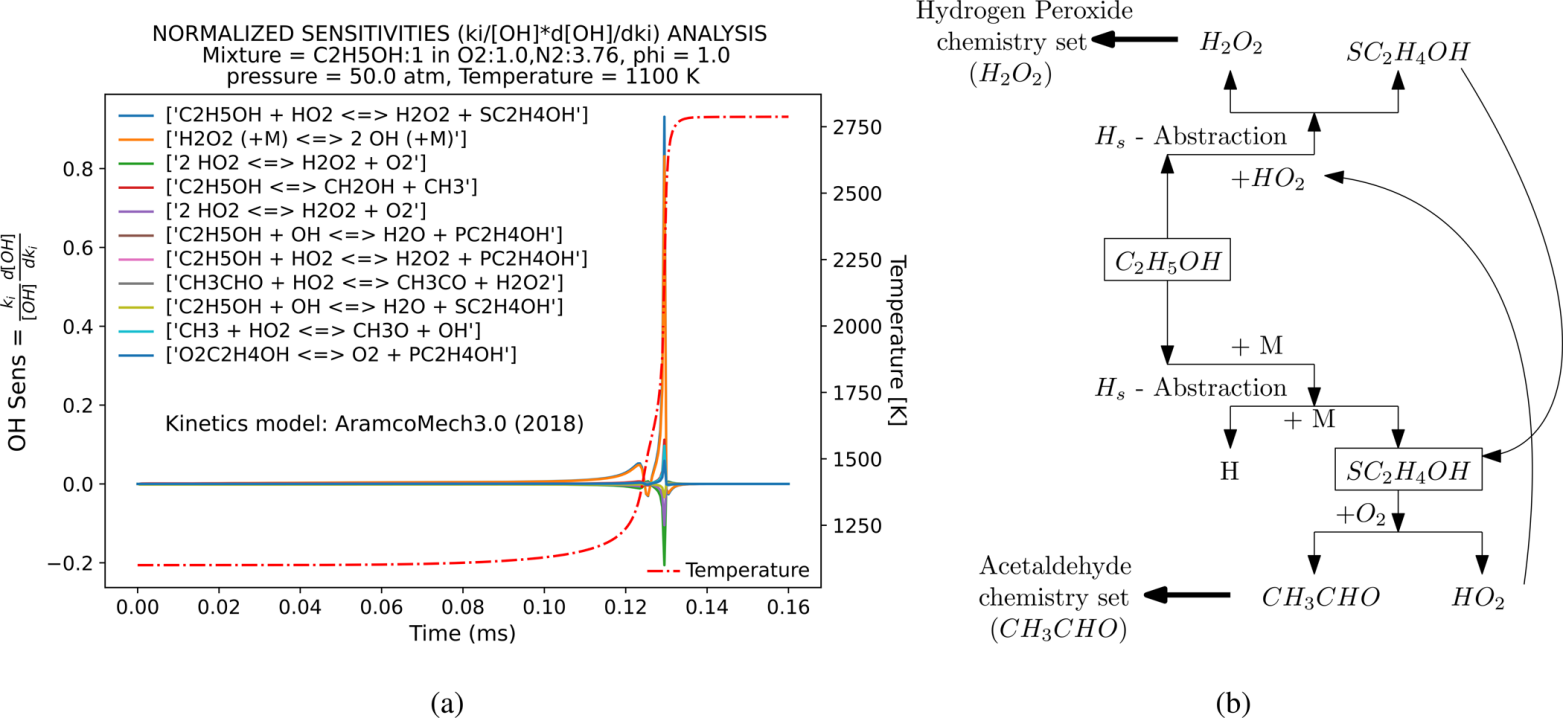
IDT sensitivity analysis of an Ethanol/Air stoichiometric mixture (ϕ = 1.0) using [OH] from the base mechanism (AramcoMech3.0) at 50 atm. (a) Exhibits the integral form of the sensitivity analysis of the first 11 reactions on the OH radical concentration as a function of time from the “start” (1100 K) up to the “ignition point”, as shown in the dotted-dashed red line temperature profile. (b) shows the main ethanol oxidation route at high pressure (50 atm) for the base mecanism (AramcoMech3.0).

Furthermore, the reduction of the ethanol base model was executed under specific operational parameters for a 0D constant-volume IDT, considering two equivalence ratios: lean (ϕ = 0.50) and stoichiometric mixture (ϕ = 1.0) across initial temperatures of 700–1280 K at 10, 30, 50, and 80 atm. The reduction procedure involved iteratively eliminating reactions and species within the DRGEP framework until a predefined IDT threshold of 1*%* error was attained under the overall conditions. The resultant ethanol-reduced kinetic model comprises 53 species and 385 reactions, almost ten times smaller than the base mechanism. All of the mechanisms used in the reduced process are summarized in Table 3.

**Table 3:** Ethanol Mechanisms^a^

name	species	reaction	type	ref
ethanol base M. (BM)	581	3037	detailed	Metcalfe et al.^28^
ethanol reduced M. (RM)	53	385	reduced	

a BM, Base mechanism; RM, Reduced mechanism.

### 2.3 Comparison between the Reduced and the Base Kinetics Mechanism

#### 2.3.1 Ignition Delay Times (IDT)

Figure 3 shows IDT simulations of the mixture of ethanol/air comparing the results using the ethanol reduced mechanism (RM) (“Ethanol RM_1st”) (− · · −) with the base mechanism (Ethanol BM)^28^ (−). The simulations were performed for lean, stoichiometric, and rich combustion conditions (ϕ = [0.5, 1.0, 1.5]) at pressures of 1, 10, and 100 atm and initial temperatures ranging from 700 to 1250 K. The maximum difference between the results obtained using the reduced and base mechanisms in the IDT simulation was 0.8%, which is within the predefined error threshold of the DRGEP method.

**Figure 3:**
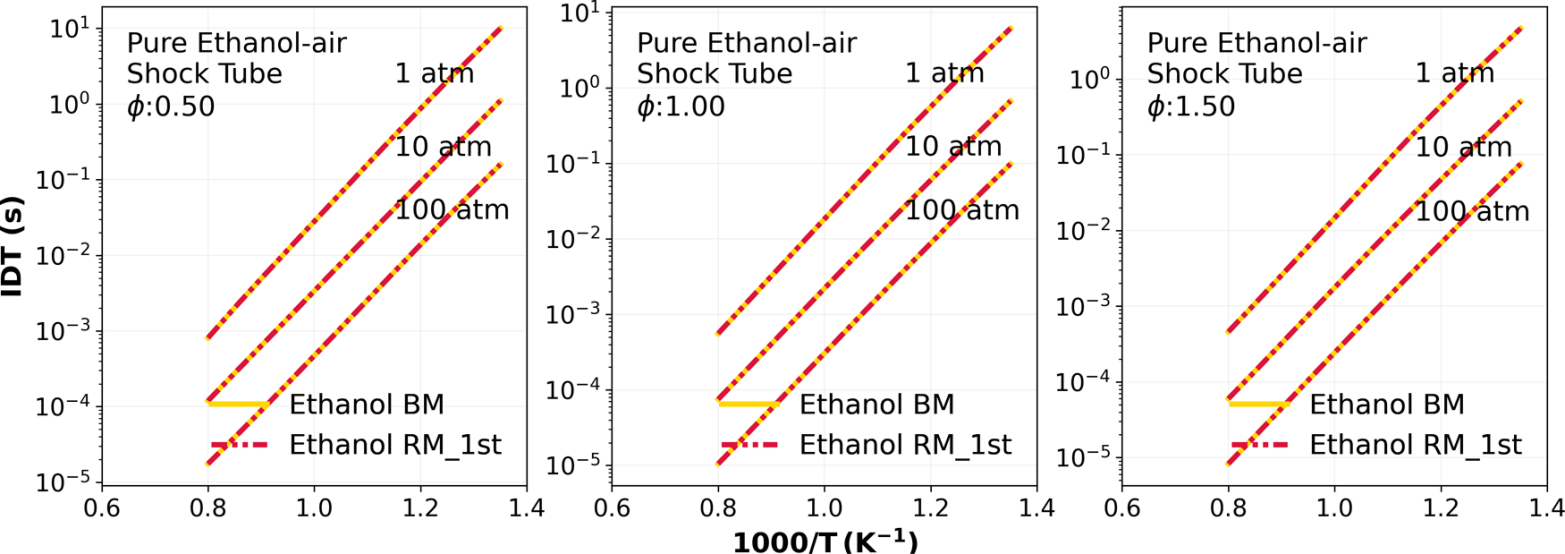
Numerical ethanol IDT comparison between the ethanol reduced mechanism (RM) (“Ethanol RM_1st”) (red dashed-double dotted lines) and the ethanol BM mechanism (AramcoMech3.0) (yellow dashed line) from Metcalfe et al.^28^ at pressures of 1, 10, 100 atm, and considering lean (ϕ = 0.5), stoichiometric (ϕ = 1.0), and rich conditions condition. The simulations were performed using an ideal EoS.

Despite the good agreement between the ethanol reduced (RM) (“Ethanol RM_1st”) and the ethanol BM mechanism, they both present a considerable deviation from the experimental high-pressure shock tube data of Cancino et al.^25^ at *T* ≤ 1100 K and 50 atm (square), as shown in Figure 1. To handle this issue, a straightforward approach consists of modifying the reaction rate of the key reactions from the IDT sensitivity analysis performed in Figure 3a by replacing its constant reaction rate (*k*) with another one from the literature under the target conditions. Increasing or decreasing the reactivity of each key reaction was done by changing its pre-exponential (*A*), activation energy (*E*_a_), and temperature exponent (*b*) of the Arrhenius equation, as summarized in Table 4. The *k* constant improvement methodology has been used in the literature by Mittal et al.,^40^ Song et al.,^41^ Roy and Askari,^15^ and Plácido et al.^20^

**Table 4:** Modifying “Ethanol RM_1st” Key Reaction Constant Rate (*k*) To Improve IDT Simulations at *T* ≤ 1100 K and 50.0 atm

reaction	previous (*k*)	modified (*k*)
Ethanol (C_2_H_5_OH) 1100 K and 50 atm		
R. [264] C_2_H_5_OH + HO_2_ ⇌ H_2_O_2_ + SC_2_H_4_OH	{*A*: 2.45 × 10^–05^, *b*: 5.26, *E*_a_: 7475.1}	{*A*: 0.028, *b*: 4.32, *E*_a_: 8530.0}^7^
R. [264] C_2_H_5_OH + HO_2_ ⇌ H_2_O_2_ + SC_2_H_4_OH	—/—	{*A*: 1.95 × 10^+24^, *b*: −4.97, *E*_a_: 0.0}^25^
type: added duplicated		

The improved ethanol reduced mechanism (called Ethanol RM_2nd) (green dashed lines) in Figure 4 presents a very good agreement with the stoichiometric ethanol experimental data reported earlier^25^ at 50 atm in comparison with the ethanol BM mechanism (−), and the first ethanol reduced “Ethanol RM_1st” (red dashed-double dotted lines).

**Figure 4:**
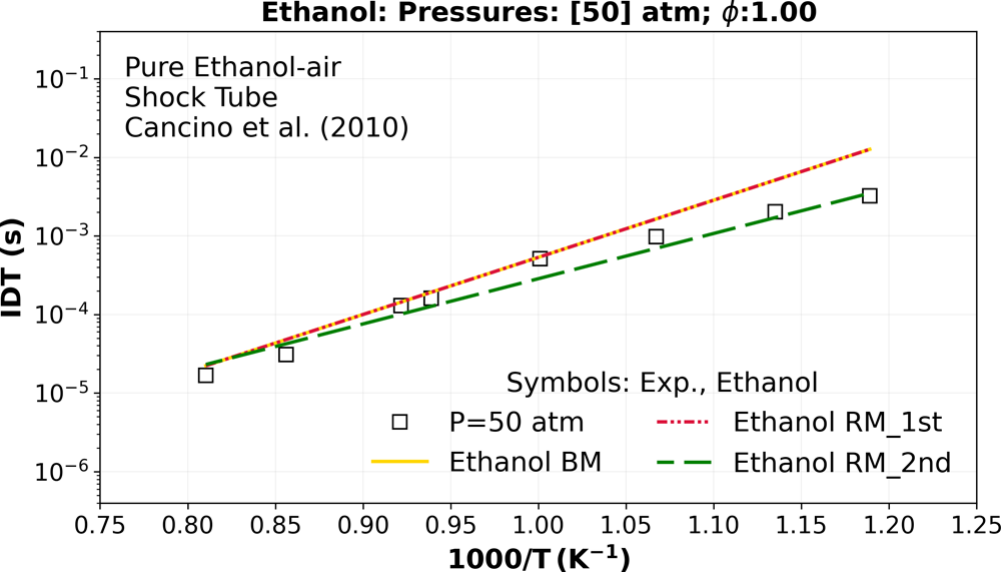
Numerical stoichiometric IDT simulations comparing the ethanol reduced mechanism (RM) (“Ethanol RM_1st”) (red dashed-double dotted lines), the ethanol BM (AramcoMech3.0)^28^ (yellow dashed lines), and the Ethanol RM_2nd (green dashed lines) versus the experimental high-pressure shock tube data of Cancino et al.^25^ at 50 atm (□).

#### 2.3.2 Laminar Flame Speed (LFS)

As reported in Section 2.2, the reduction process was exclusively performed using IDT simulations. No LFS simulations were introduced or used there due to the high computational cost in the reduction process, as the base mechanism (AramcoMech3.0)^28^ comprises more than five hundred species and more than three thousand reactions, as indicated in Table 3.

Despite this, Figure 5a shows LFS simulations using the ethanol reduced mechanism (RM) (“Ethanol RM_1st”) (red dashed-double dotted lines) and the base mechanism (“Ethanol BM”) (AramcoMech3.0)^28^ (yellow dashed lines). These simulations were conducted at 450 K initial temperature, at pressures of 2 atm (diamond) and 4 atm (square), and equivalence ratios ranging from ϕ = 0.7 to ϕ = 1.4, matching the LFS ethanol experimental conditions from Hinton et al.^42^ The numerical results indicate very good agreement at lean (ϕ < 0.90) and rich (ϕ > 1.1) conditions between the “Ethanol RM_1st” and the “Ethanol BM”, but they exhibit a maximum relative deviation of approximately 2.1% at stoichiometric ϕ = 1.0 condition, as expected due to the absence of LFS consideration in the reduction process. Additionally, both models notably deviate from the experimental LFS data of Hinton et al.,^42^ particularly at lean and stoichiometric conditions.

**Figure 5:**
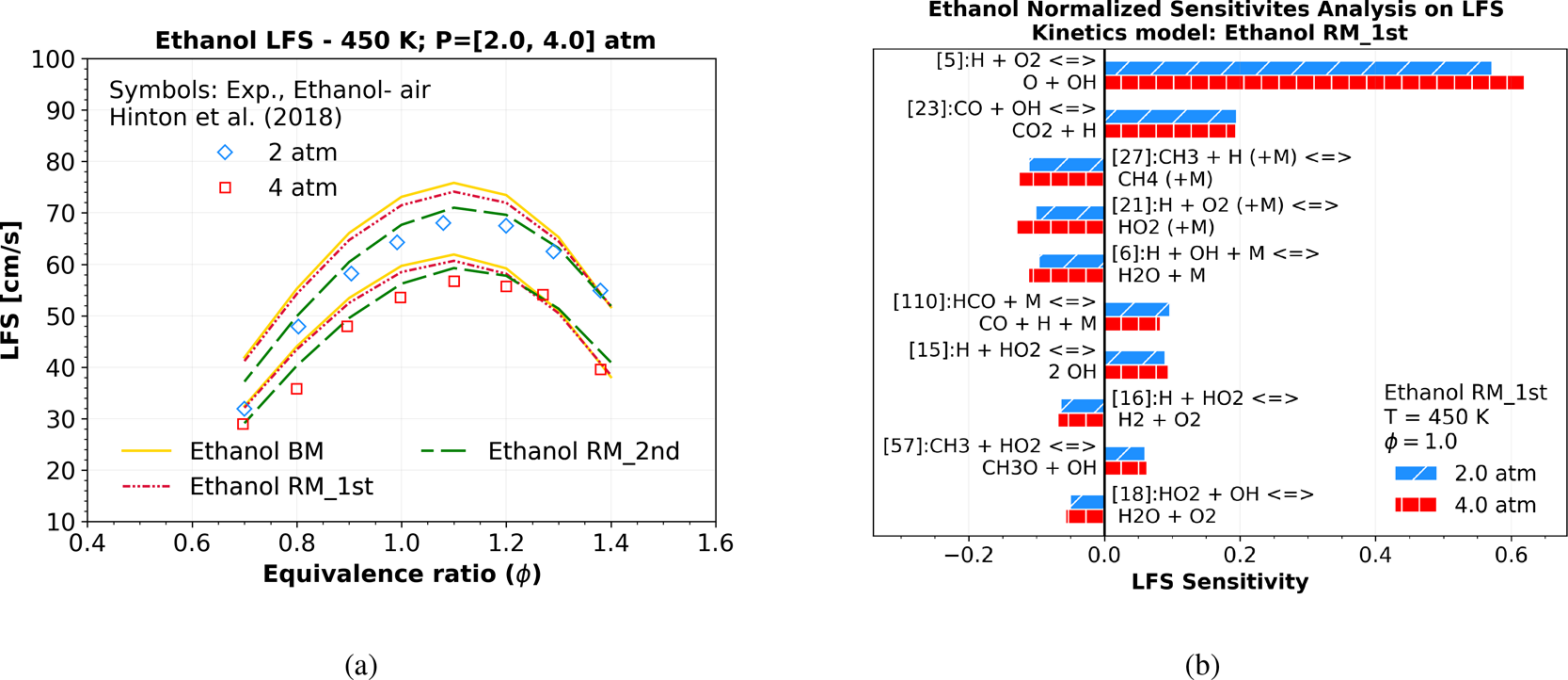
(a) Numerical stoichiometric LFS simulation using ideal EoS at 450 K and 2 and 4 atm pressure considering the “Ethanol RM_1st” mechanism (red dashed-double dotted lines), the improved reduced mechanism called by Ethanol RM_2nd (green dashed lines) and the Ethanol BM (yellow). All of them were compared with Hinton et al.^42^ LFS experimental data. Additionally, (b) shows an LFS sensitivity analysis at 450 K, 2, and 4 atm conditions, the same experimental condition of Hinton et al.^42^

A laminar flame speed (LFS) sensitivity was performed to improve the “Ethanol RM_1st” LFS simulations regarding the previous conditions of Hinton et al.'s^42^ experimental data, as illustrated in Figure 5b. This improvement was reached by modifying the reaction rate of each selected key reaction by replacing its constant reaction rate (*k*) with another one from the literature, as done in Section 2.3.1.

All the modified elementary key reactions constant rates (*k*) are numbered as **R. (5), R. (23), R. (27), R. (21),** and **R. (110)**, while the maintained key reaction constant rate (*k*) are numbered as **R. (6), R. (15), R. (16), R. (57),** and **R. (18)** in the mechanism, as observed in Figure 5b. Furthermore, these modifications are based on previous hydrogen and ethanol kinetics mechanisms available in the literature^7,36,40,43^ as summarized in Table 5.

**Table 5:** Modifying Ethanol Key Reactions Constant Rate (*k*) of “Ethanol RM_1st” To Improve LFS Simulations at 450 K and [2.0, 4.0] atm Conditions^a^

reaction	previous (*k*)	modified (*k*)	ref
**R**. (5) H + O_2_ ⇌ O + OH	{*A*: 1.04 × 10^+14^, *b*: 0.0, *E*_a_: 1.53 × 10^+04^}	{*A*: 9.65 × 10^+14^, *b*: −0.262, *E*_a_: 1.62 × 10^+04^}	^7^
**R**. (23) CO + OH ⇌ CO_2_ + H	{*A*: 7.02 × 10^+04^, *b*: 2.05, *E*_a_: −355.7}	{*A*: 6.34 × 10^+04^, *b*: 2.05, *E*_a_: −355.67}	^7,36^
**R**. (27) CH_3_ + H (+M) ⇌ CH_4_ (+M) type: falloff	high-P-rate-constant: {*A*: 1.27 × 10^+16^, *b*: −0.63, *E*_a_: 383.0}	high-P-rate-constant: {*A*: 6.35 × 10^+15^, *b*: −0.63, *E*_a_: 383.0}	^7,36^
**R**. (21) H + O_2_ (+M) ⇌ HO_2_ (+M) type: falloff	low-P-rate-constant: {*A*: 1.74 × 10^+19^, *b*: −1.23, *E*_a_: 0.0}	low-P-rate-constant: {*A*: 1.23 × 10^+19^, *b*: −1.23, *E*_a_: 0.0}	^43^
**R**. (110) HCO (+M) ⇌ H + CO (+M) type: three-body	{*A*: 5.70 × 10^+11^, *b*: 0.66, *E*_a_: 1.49 × 10^+04^}	{*A*: 4.75 × 10^+11^, *b*: 0.66, *E*_a_: 1.49 × 10^+04^}	^43^

a ref, Reference.

Figure 5a also shows the LFS simulation at the same operating experimental conditions of Hinton et al.,^42^ but after modifying the constant rate (*k*) of some key reactions. It is possible to notice better agreement between the ethanol-reduced M. resulted (called Ethanol RM_2nd) (- −) and the LFS experimental data at all ranges of equivalence ratios at 2 atm (◊) and 4 atm (□).

## 3 Real Equations of State

Although the ideal state equation (EoS) is typically utilized for high-pressure combustion, it is crucial to consider a more precise gas equation of state to understand quantitatively and qualitatively the real gas effects on LFS simulations and shock tube IDT simulations.

One of the most accurate real equations of state is the multiparameter equation proposed by Span,^44^ which is based on the Helmholtz energy formulation. However, this EoS is computationally expensive due to its large number of parameters, which are available only for certain stable species. This makes this EoS unsuitable for detailed or reduced chemical models with more than a dozen species.

Therefore, in this study, two cubic multicomponent equations of state were selected to predict real gas behavior: Redlich–Kwong (R–K) and Peng–Robison (P–R). It is because they are more commonly used for modeling real gas effects, as mentioned by Green and Southard.^45^ In addition, despite being relatively less complex, cubic equations of state nonetheless have 2–3 empirical parameters. These EoS are known for their accuracy.^18,19^ Furthermore, these R–K and P–R EoS are already implemented and available in the CANTERA code.^21,46^

For mixtures, R–K ([Disp-formula eq1]) and P–R ([Disp-formula eq2]) EoS assume, respectively, the following form:

$$P=\frac{RT}{V-b_{\mathrm{mix}}^{★}}-\frac{a_{\mathrm{mix}}^{★}}{T^{1/2}V(V+b_{\mathrm{mix}}^{★})}$$ 1

$$P=\frac{RT}{V-b_{\mathrm{mix}}^{★}}-\frac{a_{\mathrm{mix}}^{★}(T)}{V^{2}+2b_{\mathrm{mix}}^{★}V-b_{\mathrm{mix}}^{★2}}$$ 2

where the coefficients *a*_mix_^★^, *b*_mix_^★^, and *a*_mix_^★^(*T*) are dependent on the mole fraction of each species *i* (*X*_*i*_) and *j* (*X*_*j*_) in the mixture, as indicated in eqs 3–5.

$$a_{\mathrm{mix}}^{★}=\sum_{i}\sum_{j}X_{i}X_{j}a_{i,j}^{★}=\sum_{i}\sum_{j}X_{i}X_{j}\sqrt{a_{i}^{★}a_{j}^{★}}$$ 3

$$b_{\mathrm{mix}}^{★}=\sum_{i}X_{i}b_{i}$$ 4

$$a_{\mathrm{mix}}^{★}(T)=\sum_{i}\sum_{j}X_{i}X_{j}a_{i,j}^{★}(T)=\sum_{i}\sum_{j}X_{i}X_{j}\sqrt{a_{i}^{★}a_{j}^{★}}\sqrt{\alpha_{i}\alpha_{j}}$$ 5

$$\alpha =(1+\kappa (1-\sqrt{T/T_{c}}{)}^{2}$$ 6

$$\kappa =0.37464+1.54226\omega -0.26992\omega^{2}$$ 7

Here, the influence of molecular interactions is given by the species van der Waals repulsive volume correction (*b* ^★^) parameter and the attraction (*a* ^★^) parameter, presented in eqs 8 and 9, where *R* is the gas constant (8.314 J mol^–1^ K^–1^); V is the molar volume (m^3^ mol^–1^); *P* is the pressure (Pa); *T* is the absolute temperature (*K*); *P*_c_ is the critical pressure for the component of interest (Pa); *T*_c_ is the critical temperature for the component of interest (K). In addition to P–R EoS, it is necessary to obtain the temperature correlation, represented by the symbol alpha (α), and also the parameter kappa (κ) generalized with omega (ω), which is the acentric factor specified to the interest component. (ω) measures the nonsphericity of the species molecules, Green and Southard.^45^

$$a^{★}=0.42748\frac{R^{2}T_{c}^{5/2}}{P_{c}}$$ 8

$$b^{★}=0.08664\frac{RT_{c}}{P_{c}}$$ 9

The parameters (*a* ^★^) and (*b* ^★^) in eqs 8 and 9 need to be estimated for all species included in the chemical kinetics model. These parameters are derived from the critical properties of the species, such as pressure (*P*_c_) and temperature (*T*_c_), which are typically available for almost all stable species. However, they may not be accessible for many radicals and intermediate species in most kinetic mechanisms. Therefore, the critical properties of species in the “Ethanol RM_2nd” kinetics mechanism must also be estimated. Supporting Information for all chemical species molecule structures present in the “Ethanol RM_2nd” was provided, containing the formula, structure, and the structure-based chemical identifier (InChI) from IUPAC^47^ and the InChI Trust.^48^

More information about the thermodynamics properties obtained considering the multicomponent mixture expression of the P–R and R–K cubic EoS (eqs 1 and 2) integrated into the definition of Helmholtz free energy and the chemical kinetics real gases effects on mass action kinetics and laminar flame speed are available in a second Supporting Information provided in this work.

### 3.1 Critical Property Estimation for Chemical Species

In the present study, critical pressure (*P*_c_), boiling point (*T*_b_), and critical temperature (*T*_c_) of each species present in the “Ethanol RM_2nd” model were estimated using the Joback group contribution method,^49^ as these values were not available in the literature. The Joback method, widely employed in chemistry, considers the fundamental structural properties of individual chemical groups within each species.^13,23^ This methodology computes various compound properties by considering the recurrence in the molecule of each group multiplied by its respective contribution, which in turn depends on structurally intrinsic parameters of bonds. It assumes that group interactions are insubstantial and apply to nonpolar and polar species. In the works of Owczarek and Blazej,^50^ critical temperatures (*T*_c_) for various gases were presented using multiple methodologies, and the Joback method demonstrated an error limitation of less than 10.0% for both unbranched and branched hydrocarbons.

The boiling point (*T*_b_) of a species can be estimated by employing the Joback method with the following equation:

$$T_{b}\;(K)=198+\sum_{j}N_{j}C_{T_{b},j}$$ 10

Afterward, the critical pressure (*P*_c_) and temperature (*T*_c_) can be estimated by

$$P_{c}\;(\mathrm{bar})={\left[0.113+0.0032N_{\mathrm{atoms}}-\sum_{j}N_{j}C_{p_{c},j}\right]}^{-2}$$ 11

$$\frac{T_{c}}{T_{b}}={\left[0.584+65\left\{\sum_{j}N_{j}C_{T_{c},j}\right\}-{\left\{\sum_{j}N_{j}C_{T_{c},j}\right\}}^{2}\right]}^{-1}$$ 12

In these equations, *j* represents the bond chain type of group, and *N*_*j*_ is the total number of *j* groups in the analyzed species. It is worth noting that each group type *j* contributes to the critical temperature (*C*_*T*_c_,*j*_), boiling point temperature (*C*_*T*_b_,*j*_), and critical pressure (*C*_*p*_c_,*j*_). The term *N*_atoms_ denotes the quantity of atoms in the species analyzed. The values of each group contributions (*C*_*T*_c_,*j*_, *C*_*T*_b_,*j*_, *C*_*p*_c_,*j*_) can be obtained from Poling et al.^13^ Additionally, it is important to mention that group contribution data for radicals and short-lived species are generally unavailable. Nonetheless, the parameters for unstable species can usually be considered equivalent to those of similar stable species, as reported by Plácido et al.,^51^ Kogekar et al.,^21^ and Tang and Brezinsky.^52^ A comparison between the literature ethanol^13,23^ and Joback’s estimated critical properties is presented in Table 6. Also, the ethanol chemical structure is illustrated in Figure 6.

**Table 6:**
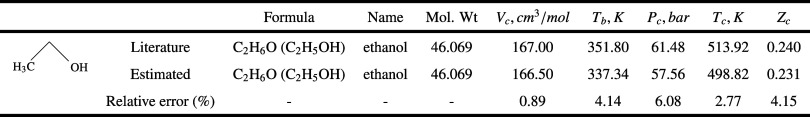
Literature Values for Ethanol Properties^13,23^ versus Estimated Critical Properties by Joback’s Group Method

**Figure 6:**
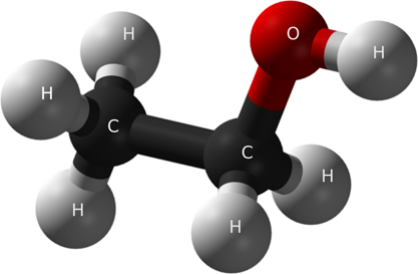
3D structure of ethanol: a molecular model where white spheres represent hydrogen atoms, black spheres carbon atoms, and red spheres oxygen atoms. This illustration was created based on molecular representations available in previous literature.^53^

After this process, the parameters (*a* ^★^), (*b* ^★^), and ω from R–K and P–R EoS for each species were implemented directly into the developed “Ethanol RM_2nd” reduced chemical kinetics mechanism file, available as Supporting Information.

## 4 Computational Time Comparison of Different Equation of States (EoS)

A comparison of the simulations using the “Ethanol RM_2nd” model using the Ideal, R–K, and P–R EoS was performed to get the average computational time when comparing results of LFS in ranges of temperatures from 298 to 949 K, pressures from 1 to 10 atm, and equivalence ratio (ϕ) from 0.6 to 1.6. Also, the average computational time for stoichiometric ignition delay (IDT) ranged from 769 to 1430 K, and 10–80 atm were calculated.

The simulations used Cantera version 3.0.0 through a Python interface on a Linux Ubuntu 22.04.4 LTS operating system with 128.0 GB of RAM and an Intel Xeon W-2265 CPU with 24 processors. LFS was calculated using two different grid refinement criteria: medium and fine. The medium grid refinement utilized a 0.03 m width and refinement criteria with a curve of 0.25, a slope of 0.06, and a ratio of 3. The fine grid refinement employed a 0.01 m width and refinement criteria, including a curve of 0.12, a slope of 0.008, and a ratio of 3. The time taken for each EoS in the “Ethanol RM_2nd” model was determined with 60 data points for the IDT calculation and around 33 data points for LFS calculations, and the time for each data point was recorded. The mean time for all data points was considered to be the computational time for each EoS.

Understanding when to use or not use a real gas state equation is crucial in computational fluid dynamics (CFD). The main issue is the need for a reduced mechanism that can produce results with high accuracy and minimum computational time. The data presented in Table 7 compares the average time calculation for each equation of state normalized by the ‘Ethanol RM_2nd’ mechanism using the Ideal EoS. This information is useful for LFS and ignition delay time (IDT) simulations using the ideal EoS, offering practical guidance for future research and applications about the computational costs of using a more accurate equation of state (RK or PR EoS) at low or intermediate pressure and temperature conditions.

**Table 7:** Mean Computational Time Requisite for Each Equation of State (EoS) in the “Ethanol RM_2nd” Model and the Normalized Ratio of Time Concerning the “Ethanol RM_2nd” Using Ideal Gas

LFS			IDT		
EoS to LFS	*t* (s)	*t*/*t*_ideal_	EoS to IDT	*t* (s)	*t*/*t*_ideal_
ideal EoS	47.23	1.00	ideal EoS	0.18	1.00
R–K EoS	320.73	6.79	R–K EoS	1.25	6.93
P–R EoS	422.53	8.95	P–R EoS	2.90	16.04

As expected and shown in Table 7, the Peng–Robinson equation of state (PR EoS) requires higher computational resources compared to the Redlich–Kwong state equation (RK EoS) due to the inclusion of an additional parameter (the acentric factor (ω) presented in the real equations of state section). This parameter needs to be used along with the molecular interaction parameters (*a* ^★^) and (*b* ^★^), providing more accurate results for PR EoS compared to RK EoS. On the other hand, RK EoS only requires the latter two molecular interaction parameters.

## 5 Results and Discussion

### 5.1 IDT and LFS Validations for Subcritical, Transcritical, and Supercritical Combustion Conditions Using Real Gas State Equations

#### 5.1.1 Anhydrous Ethanol IDT

Experimental high-pressure shock tube (ST) data conditions were compared with the developed ethanol-reduced mechanism under more comprehensive high-pressure and temperature conditions to test its accuracy. These experimental shock tube (ST) data for ethanol were collected from.^25,35,54^ It is important to point out that the supercritical pressure of ethanol/air mixtures is over 73 atm, as presented in previous works of Plácido et al.^20,51^

Figure 7 shows a good agreement between the transcritical numerical IDT simulations and the experimental data from,^25^ respectively, with a root mean square error (RMSE) of all three EoS quite similar exhibiting values around 6.210 × 10^–04^ (s) at 10 (□), around 1.762 × 10^–03^ (s) at 30 (diamond), and of around 9.200 × 10^–05^ (s) at 50 atm (circle).

**Figure 7:**
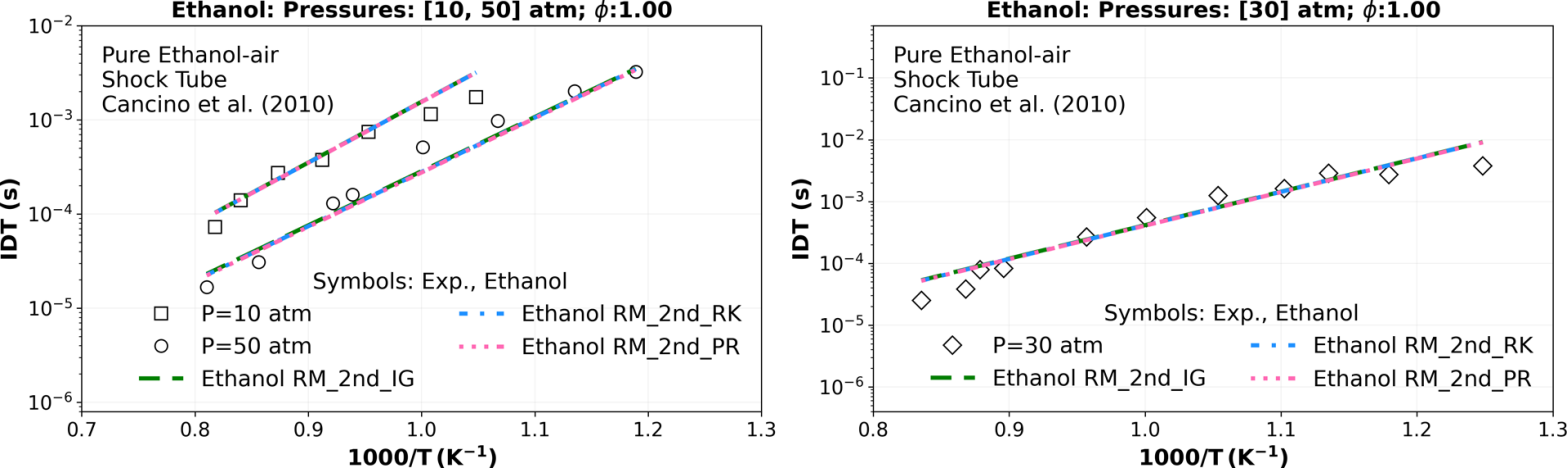
Numerical stoichiometric IDT transcritical simulations of a mixture composition of ethanol, O_2_, and N_2_ comparing the “Ethanol RM_2nd” using ideal gas EoS (_IG) (green dashed lines), R–K EoS (_RK) (blue dashed dotted line), and P–R EoS (_PR) (pink dotted dashed lines) versus the experimental high-pressure shock tube data of Cancino et al.^25^ at 10 (square), 30 (diamond), and 50 atm (circle).

Concerning the same conditions, a relative deviation around 0.71% at 10 atm, 2.15% at 30 atm, and 3.62% at 50 atm is observed between P–R EoS and the ideal EoS as inserted in the last column in Table 8. Regarding R–K EoS, the relative deviations were 0.45, 1.35, and 2.36% for the ideal EoS at the same experimental pressure data. There was no significant difference between the ideal gas state equation and the two cubic EoS used at these transcritical conditions (10, 30, and 50 atm), not justifying the higher computational cost in IDT for the real gas R–K EoS (seven times higher) and for the P–R EoS (16 times higher), as indicated in Table 7.

**Table 8:** IDT RMSE of the “Ethanol RM_2nd” Model against Various High-pressure Shock Tube Ethanol Experimental Data^a^,^b^

	experiments		kinetics model				
figure	authors	pressure (atm)	ideal gas (s)	real gas (R–K) (s)	real gas (P–R) (s)	rel. D. (R–K) (%)	rel. D. (P–R) (%)
Ethanol							
Figure 7	^25^	10	6.210 × 10^–04^	6.170 × 10^–04^	6.130 × 10^–04^	0.456	0.715
Figure 7	^25^	30	1.762 × 10^–03^	1.750 × 10^–03^	1.720 × 10^–03^	1.352	2.147
Figure 7	^25^	50	9.200 × 10^–05^	8.200 × 10^–05^	6.900 × 10^–05^	2.357	3.617
Figure 8	^54^	13	2.370 × 10^–04^	2.310 × 10^–04^	2.250 × 10^–04^	0.666	0.932
Figure 8	^54^	20	1.091 × 10^–03^	1.079 × 10^–03^	1.054 × 10^–03^	1.017	1.412
Figure 8	^54^	40	1.560 × 10^–04^	1.650 × 10^–04^	1.730 × 10^–04^	1.854	2.887
Figure 8	^54^	75	1.004 × 10^–03^	9.860 × 10^–04^	9.280 × 10^–04^	2.960	5.099
Figure 9	^26^	80	7.440 × 10^–04^	7.320 × 10^–04^	6.980 × 10^–04^	3.160	5.392
Figure 9		250				11.237	17.248
Figure 9		500				24.140	33.236
Figure 9		1000				49.865	59.192

a In addition, the relative deviations (%) of IDT simulations were analyzed using distinct cubic equations of states (Redlich–Kwong and Peng–Robinson) compared to the IDT simulations from the ideal EoS under the same pressure conditions.

b Rel. D., Relative deviation about the IDT simulations adopting the ideal EoS.

In Figure 8, the “Ethanol RM_2nd” mechanism shows a good agreement with the ignition delay time (IDT) transcritical shock tube data from Heufer et al.^54^ at various pressures. The RMSE is approximately 2.370 × 10^–04^ (s) at 13 atm, 1.091 × 10^–03^ (s) at 20 atm, and 1.560 × 10^–04^ (s) at 40 atm. Additionally, compared with the supercritical shock tube data, the RMSE is about 1.004 × 10^–03^ (s) at 75 atm for all tested equations of state (ideal EoS, R–K EoS, and P–R EoS). At transcritical and supercritical conditions, a relative deviation of approximately 0.94% at 13 atm, 1.41% at 20 atm, 2.89% at 40 atm, and 5.09% at 75 atm is observed between the P–R EoS and the ideal EoS. Relative deviations using the R–K EoS are approximately 0.7, 1.02, 1.85, and 3.00%, respectively, at the same experimental pressures.

**Figure 8:**
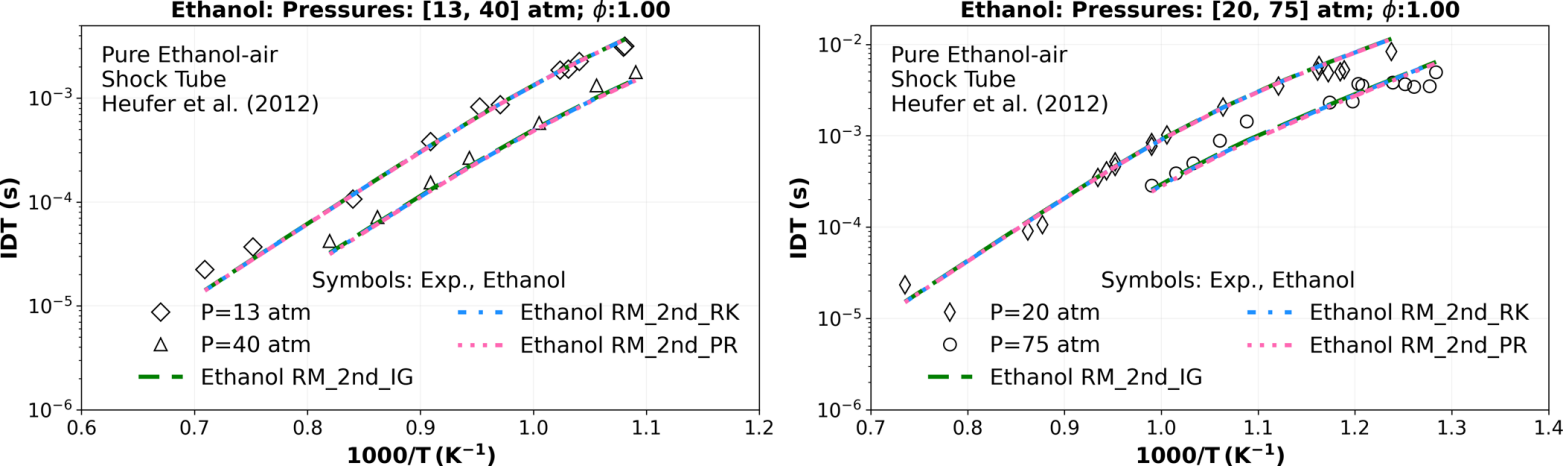
Numerical stoichiometric IDT simulations comparing the “Ethanol RM_2nd” using ideal gas EoS (_IG) (green dashed lines), R–K EoS (_RK) (blue dashed dotted lines), and P–R EoS (_PR) (pink dotted dashed lines) versus the experimental high-pressure shock tube data of Heufer et al.^54^ at 13 (◊), 20 (◊), 40 (△) and 75 atm (○).

At 10, 13, 20, 40, and 50 atm (for transcritical conditions), no significant difference (deviation < 5%) was observed between the ideal gas equation of state (EoS) simulations and the real gas EoS simulations. However, at 75 atm, an initial nonideal behavior is noticed (deviation ≥ 5%), suggesting that higher pressures increase the deviation between the ideal gas and real gas EoS. Indeed, in Figure 9, an anhydrous ethanol/air mixture is compared to supercritical shock tube experimental data from Lee et al.^26^ at 80 atm, showing a 5.4% relative deviation from the P–R EoS to the ideal gas EoS, and a 3.16% relative deviation from the R–K EoS to the ideal gas EoS.

**Figure 9:**
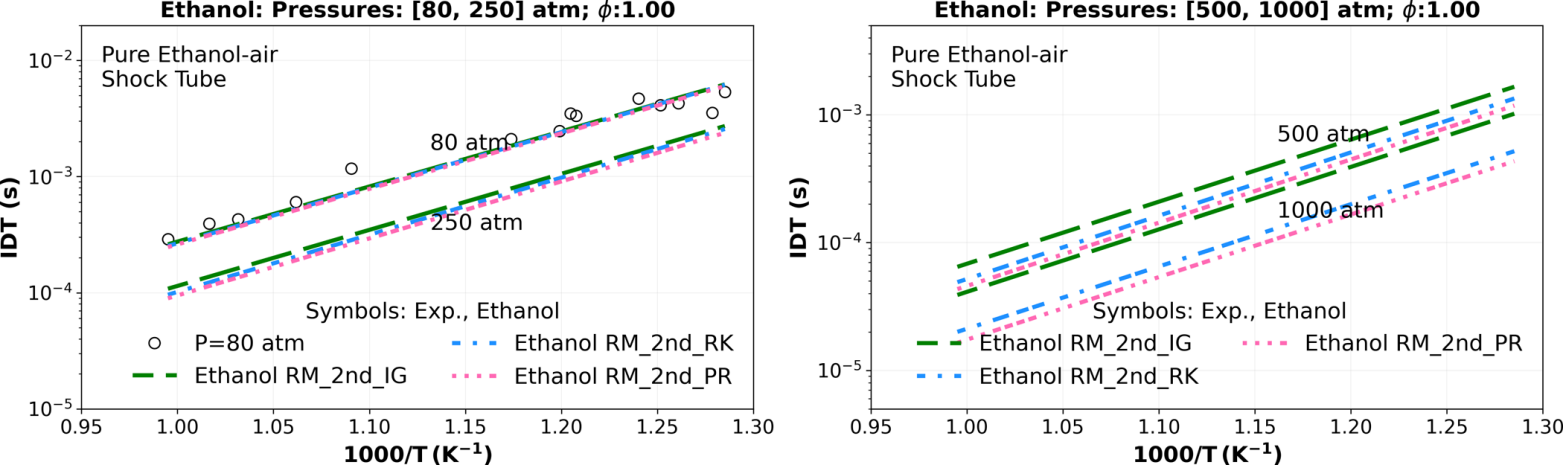
Numerical stoichiometric IDT supercritical simulations of a mixture composition of Ethanol, O_2_, and N_2_ comparing the “Ethanol RM_2nd” using ideal gas EoS (_IG) (green dashed lines), R–K EoS (_RK) (blue dashed dotted lines), and P–R EoS (_PR) (pink dotted dashed lines) at 250 atm, 500 and 1000 atm, and also versus the experimental high-pressure shock tube data of Cancino et al.^25^ at 80 atm (circle).

Considering the absence of shock tube IDT experimental data for an anhydrous ethanol/air mixture at pressures exceeding 100 atm and in light of the prior results, Figure 9 includes three additional supercritical IDT simulations at 250, 500, and 1000 atm, highlighting the differences when simulating ideal and real gas EoS (R–K and P–R EoS). It is found that increased pressure (250–1000) significantly accentuates the deviation from the ideal EoS to R–K EoS and P–R EoS, resulting in 11.23 and 17.25% deviation at 250 atm, 24.14 and 33.24% at 500 atm, and 49.87 and 59.19% at 1000 atm. These values are summarized in the last two columns of Table 8. Finally, the computational cost associated with using real gas state equations (R–K and P–R EoS) in IDT simulations, as indicated in Table 7, is justified by the substantial deviation between these real gas EoS and the ideal gas EoS.

Table 8 estimates the agreement of the “Ethanol RM_2nd” mechanism with the ethanol IDT measurements at distinct pressures based on the RMSE values. Furthermore, the relative deviations (%) of IDT simulations utilizing cubic state equations (Peng–Robinson and Redlich–Kwong) were compared to simulations using an ideal EoS at the same conditions, as presented in the last two columns of Table 8.

#### 5.1.2 Ethanol LFS

Unfortunately, experimental data of laminar flame speed (LFS) are unavailable in the literature under supercritical conditions (*P* > *P*_c_) and (*T* > *T*_c_). Therefore, to validate the kinetics model for a satisfactory range, different temperatures (298–949 K), compositions (anhydrous ethanol and hydrous ethanol (Supporting Information)), and pressure conditions (1 ≥ *P* ≤ 10 atm) are considered at subcritical and transcritical conditions using the Ideal gas, Peng–Robinson, and Redlich–Kwong EoS.

In the study of anhydrous ethanol fuel, the results presented in Figure 10 at atmospheric pressure (1 atm) and temperatures of 298, 358, and 398 K exhibit a good agreement with most of the experimental data from various sources found in the literature, such as Gülder,^55^ Egolfopoulos et al.,^56^ Bradley et al.,^57^ Liao et al.,^58^ Van Lipzig et al.,^59^ Konnov et al.,^27^ Dirrenberger et al.,^60^ Monteiro et al.,^61^ and Alviso et al.^62^ This indicates that the “Ethanol RM_2nd” mechanism offers reliable results at atmospheric pressure, with the highest RMSE of approximately 2.2 cm/s using all three equations of state (ideal, P–R, and R–K EoS).

**Figure 10:**
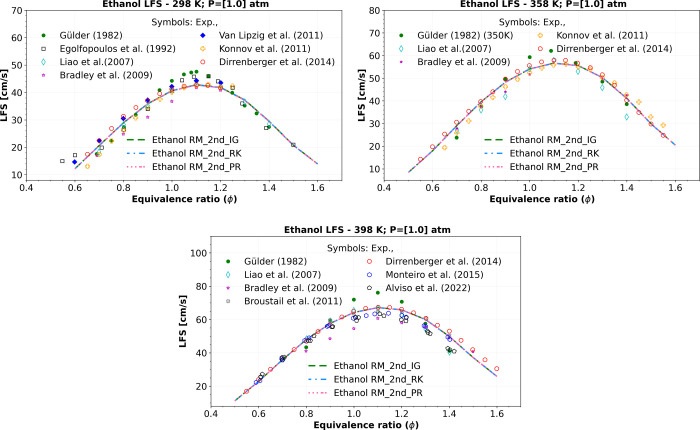
Numerical atmospheric LFS simulations of a mixture composition of ethanol, O_2_, and N_2_ comparing the “Ethanol RM_2nd” using ideal gas EoS (_IG) (green dashed lines), R–K EoS (_RK) (blue dashed dotted lines), and P–R EoS (_PR) (pink dotted dashed lines) at 298, 358, and 398 K, versus symbols representing experiments of LFS data from Gülder,^55^ Egolfopoulos et al.,^56^ Liao et al.,^58^ Bradley et al.,^57^ Van Lipzig et al.,^59^ Konnov et al.,^27^ Dirrenberger et al.,^60^ Monteiro et al.,^61^ and Alviso et al.^62^

Moreover, there is a slight difference between the three equations of state, with the highest relative deviation around 0.2%, as presented in Table 9. This underscores the prevalence of ideal gas behavior at atmospheric pressure, supporting the use of the ideal gas EoS. Furthermore, the increase in computational cost when using R–K and P–R EoS at this atmospheric pressure does not seem justified.

**Table 9:** LFS RMSE of the “Ethanol RM_2nd” Model against Various Anhydrous Ethanol LFS Experimental Data^a^,^b^

	experiments		kinetics model					
figure	authors	pressure (atm)	temp. (K)	ideal gas (s)	real gas (R–K) (s)	real gas (P–R) (s)	rel. D. (R–K) (%)	rel. D. (P–R) (%)
Anhydrous Ethanol LFS								
Figure 10a	^60^	1	298	1.320	1.354	1.363	0.060	0.076
Figure 10b	^60^	1	358	1.619	1.630	1.633	0.182	0.226
Figure 10c	^60^	1	398	2.248	2.269	2.274	0.128	0.157
Figure 11a	^42^	2	380	2.781	2.726	2.713	0.221	0.273
Figure 11a	^42^	4	380	2.360	2.334	2.329	0.472	0.576
Figure 11b	^42^	2	450	2.986	2.971	2.966	0.105	0.124
Figure 11b	^42^	4	450	2.490	2.461	2.454	0.275	0.315
Figure 12a	^57^	1	358	2.029	2.011	2.006	0.128	0.160
Figure 12a	^57^	5	358	2.686	2.598	2.575	0.746	0.863
Figure 12a	^57^	10	358	2.199	2.197	2.199	1.524	1.836
Figure 13a	^63^	1	500–949	9.380	9.328	9.319	0.153	0.239
Figure 13a	^58,63,64^	1	300–744	8.517	8.500	8.494	0.050	0.065
Figure 13b		50	300–744				0.940	1.516
Figure 13b		100	300–744				1.697	2.416
Figure 13b		250	300–744				2.042	2.416
Figure 13b		500	300–744				15.566	16.333

a In addition, the relative deviations (%) of IDT simulations were analyzed using distinct cubic equations of states (Redlich–Kwong and Peng–Robinson), compared to the IDT simulations from the ideal EoS under the same pressure conditions.

b Rel. D., Relative deviation about the LFS simulations adopting the ideal EoS.

Moreover, for other pressures, the LFS numerical results of the “Ethanol RM_2nd” model closely agree with the experimental data at 380 K, 2–4 atm, as shown in Figure 11a, and at 450 K and 2–4 atm, as shown in Figure 11b. The highest root-mean-square error (RMSE) is approximately 2.9 cm/s, as indicated in Table 9. It is worth noting that these LFS simulations exhibit no significant deviation from ideal gas behavior, presenting a marginal difference of 0.576*%* from the ideal Equation of State (EoS) when using the R–K or P–R EoS.

**Figure 11:**
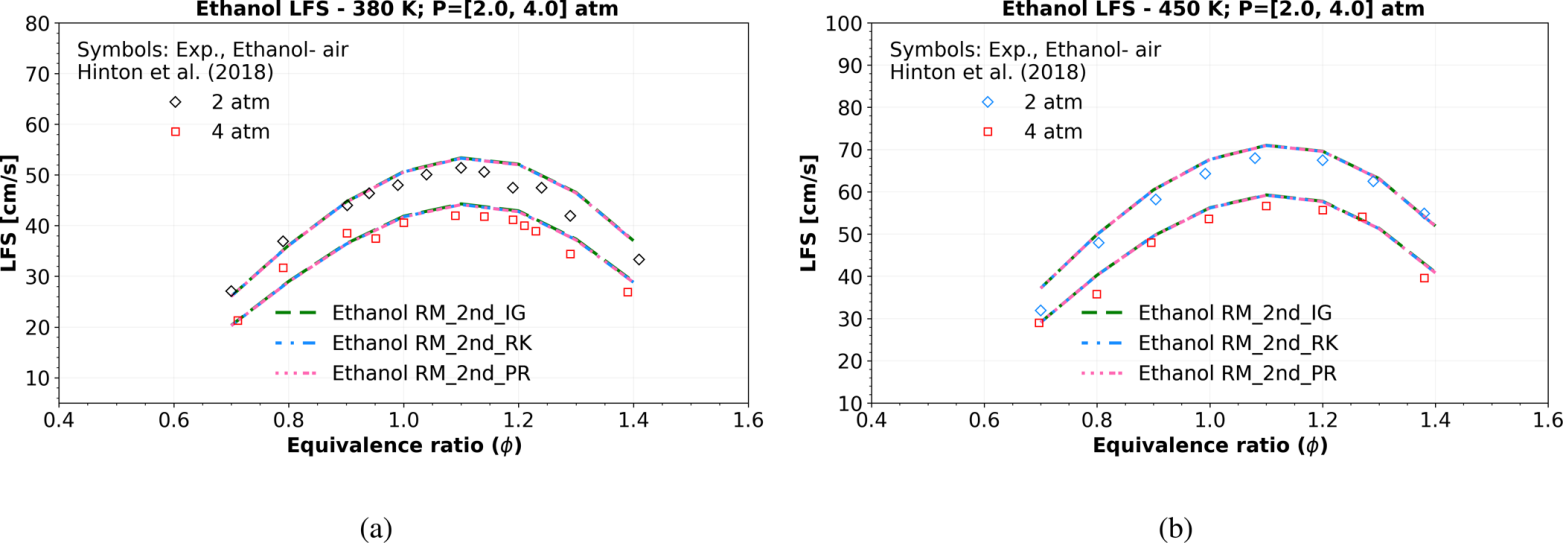
Numerical LFS simulations of a mixture composition of ethanol, O_2_, and N_2_ comparing the “Ethanol RM_2nd” using ideal gas EoS (_IG) (green dashed lines), R–K EoS (_RK) (blue dashed dotted lines), and P–R EoS (_PR) (pink dotted dashed lines) at (a) 380 K and (b) 450 K, versus symbols representing experiments of LFS data at 2 and 4 atm from Hinton et al.^42^

For pressure conditions above 4 atm, Figure 12 shows that the “Ethanol RM_2nd” model using all three EoS (Ideal, P–R, and R–K EoS) agrees well with the experimental data from Bradley et al.^57^ at 358 K, 1, 5, and 10 atm, with a root-mean-square error (RMSE) of approximately 2.5 (cm/s). Additionally, as observed in IDT simulations, it is expected that in LFS, increasing pressure (1, 5, and 10 atm) leads to an increase in the relative deviation from the ideal gas EoS to R–K (ranging from 0.128 to 1.524%) and the P–R EoS (ranging from 0.160 to 1.836%).

**Figure 12:**
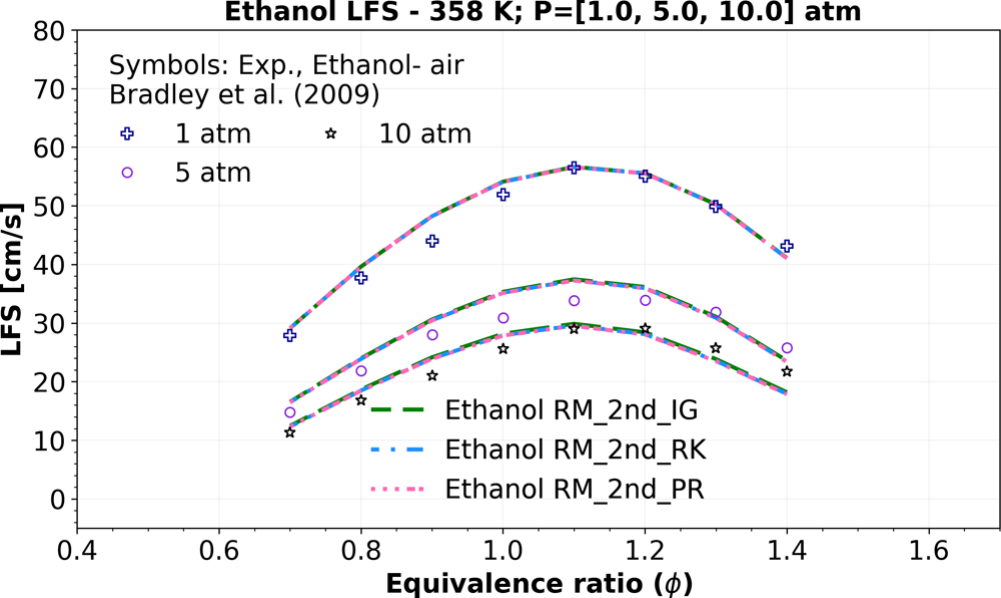
Numerical LFS simulations of a mixture composition of ethanol, *O*_2_, and *N*_2_ comparing the “Ethanol RM_2nd” using ideal gas EoS (_IG) (green dashed lines), R–K EoS (_RK) (blue dashed dotted lines), and P–R EoS (_PR) (pink dotted dashed lines) at 358 K, versus symbols representing experiments of LFS data at 1, 5, and 10 atm from Bradley et al.^57^

To observe the behavior of the “Ethanol RM_2nd” model using three different equations of state (EoS), Ideal, R–K, and P–R EoS, at temperatures above the critical temperature (*T* > *T*_c_), a recent study by Zheng et al.^63^ involved the experimental measurement of stoichiometric laminar flame speeds (LFS) in a shock tube using ethanol blends in a 21% O2–79% Ar oxidizer, also known as “airgon”, whose measurements were conducted for a 500–949 K temperature range and at atmospheric pressure. It is worth noting that the LFS experiments also involved the scaling of mixture data for fuel/argon to determine equivalent fuel/air (21% O2–79% N2) flame speeds with a maximum deviation of 1%. These values were then compared with those of our “Ethanol RM_2nd” model. The comparison, as shown in Figure 13, revealed a good agreement between the model and the experimental data, with a root-mean-square error (RMSE) of approximately 9.3 (cm/s) in an LFS range of 178–525 cm/s, representing a deviation of less than 8%.

**Figure 13:**
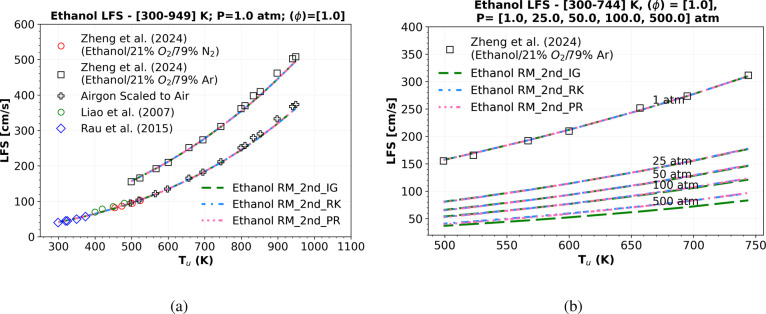
Numerical shock-tube atmospheric-pressure flame speed simulations of a mixture composition of Ethanol, O_2_, and N_2_ (air) and ethanol, O_2_, and Ar (argon) comparing the “Ethanol RM_2nd” using ideal gas EoS (_IG) (green dashed lines), R–K EoS (_RK) (blue dashed dotted lines), and P–R EoS (_PR) (pink dotted dashed lines) at (a) 1 atm versus symbols representing shock tube experiments of flame speed, in range of temperatures of 300 K up to 949 K, from Liao et al.,^58^ Rau et al.,^64^ and Zheng et al.,^63^ and (b) 1, 25, 50, 100, and 500 atm.

In the absence of LFS experimental data for an anhydrous ethanol/air mixture at pressures exceeding 15 atm, and based on previous results, Figure 13b presents four additional LFS simulations at 25, 50, 100, and 500 atm. These simulations highlight the differences between ideal and real gas EoS (P–R and R–K EoS). It is observed that as the pressure increases (100–500 atm), the relative deviation from ideal EoS to R–K EoS and P–R EoS becomes more pronounced, resulting in a 1.697 and 2.416% deviation at 100 atm, 2.042 and 2.416% at 250 atm, and 15.566 and 16.333% at 500 atm. These findings are summarized in the last two columns of Table 9. Furthermore, the computational cost of using real gas state equations (P–R and R–K EoS) in LFS simulations, as indicated in Table 7, is justified by the significant deviation between these cubic nonideal gas EoS and the ideal gas EoS.

## 6 Conclusions

The study developed a reduced kinetic model for ethanol combustion, which consisted of 53 species and 385 reactions. This model was developed to simulate subcritical, transcritical, and supercritical conditions. By using reduction techniques on the comprehensive AramcoMech3.0 mechanism, the study addressed the high computational costs associated with the detailed reaction mechanisms. Through sensitivity analysis, directed relation graph with error propagation (DRGEP), and the stoichiometric integral form of sensitivity analysis based on the concentration of OH, the reduced model effectively preserved essential pathways for high-pressure oxidation, focusing on key species such as OH, CO, HO_2_, acetaldehyde (CH_3_CHO=C_2_H_4_O), hydrogen peroxide (H_2_O_2_), and the 1-hydroxyethyl radical CH_3_CHOH.

The study’s simulations indicated that the improved reduced model (“Ethanol RM_2nd”) achieved an excellent agreement with experimental data for stoichiometric ethanol combustion in laminar flame speed (LFS) and ignition delay time (IDT) simulations at lean, stoichiometric, and rich conditions. The reduced model improved computational efficiency without compromising accuracy at pressures ranging from 1 to 80 atm and temperatures of 298–949 K.

The study also explored the limitations of the ideal gas state equation (EoS) in capturing real gas effects, particularly under ultrahigh-pressure conditions (>100 atm). By implementing the Peng–Robinson (PR) and Redlich–Kwong (RK) EoS parameters, the study addressed the discrepancies observed at pressures exceeding 100 atm. Simulations of ignition delay times at 500 atm showed that these discrepancies stayed about 24.14–33.24% for IDT and about 15.57–16.33% for LFS, respectively, for R–K and P–R EoS. The findings highlighted the importance of accurate thermodynamic modeling, with notable deviations at higher elevated pressures. It also highlights that due to the highest computational cost of PR and RK EoS, the ideal gas EoS satisfies lower and intermediate pressure ethanol combustion conditions accurately.

In summary, the developed model significantly reduces the computational cost of ethanol combustion simulations while retaining high accuracy. Incorporating real gas EoS enhances the model’s applicability to high-pressure and supercritical conditions. It is a valuable tool for future research and practical applications in combustion modeling and related technologies.

## Supplementary Material





## Notes

Thermodynamic properties of real gases; molar Helmholtz free energy and chemical kinetics of real gases; effects on mass action kinetics; hydrous ethanol laminar flame speed discussions and validations at low pressure; and structure of species comprised in the ethanol-reduced M. (Ethanol RM_2nd) (PDF)
Ethanol-reduced M. (Ethanol RM_2nd); all P–R EoS and R–K EoS intermolecular parameters (ZIP)

## References

[1] Boer, C. ; Bonar, G. ; Sasaki, S. ; Shetty, S. Application of supercritical gasoline injection to a direct injection spark ignition engine for particulate reduction. SAE Tech pap 2013.

[2] Song Y., Zheng Z., Peng T., Yang Z., Xiong W., Pei Y. (2020). Numerical investigation
of the combustion characteristics of an internal
combustion engine with subcritical and supercritical fuel. Applied Sciences.

[3] Mayer W., Schik A., Schafer M., Tamura H. (2000). Injection and mixing
processes in high-pressure liquid oxygen/gaseous hydrogen rocket combustors. J. Propul. Power.

[4] Schmitt T. (2020). Large-eddy
simulations of the mascotte test cases operating at supercritical
pressure. Flow, Turbulence and Combustion.

[5] Liu Y., Pei Y., Peng Z., Qin J., Zhang Y., Ren Y., Zhang M. (2017). Spray development and
droplet characteristics of high temperature
single-hole gasoline spray. Fuel.

[6] Li D., Yu X., Sun P., Du Y., Xu M., Li Y., Wang T., Zhao Z. (2021). Effects of
water ratio in hydrous
ethanol on the combustion and emissions of a hydrous ethanol/gasoline
combined injection engine under different excess air ratios. ACS omega.

[7] Heufer K. A., Sarathy S. M., Curran H. J., Davis A. C., Westbrook C. K., Pitz W. J. (2012). Detailed kinetic modeling study of
n-pentanol oxidation. Energy Fuels.

[8] Dagle R. A., Winkelman A. D., Ramasamy K. K., Lebarbier Dagle V., Weber R. S. (2020). Ethanol as a Renewable
Building Block for Fuels and
Chemicals. Ind. Eng. Chem. Res..

[9] Yamaguchi, A. ; Koopmans, L. ; Helmantel, A. ; Karrholm, F. P. ; Dahlander, P. Spray characterization of gasoline direct injection sprays under fuel injection pressures up to 150 MPa with different nozzle geometries. SAE Tech pap 2019.

[10] Yamaguchi A., Koopmans L., Helmantel A., Dillner J., Dahlander P. (2020). Air Motion
Induced by Ultra-High Injection Pressure Sprays for Gasoline Direct
Injection Engines. SAE Int. J. Fuels Lubr..

[11] Li X., qiang Pei Y., Qin J., Zhang D., Wang K., Xu B. (2018). Effect of ultra-high injection pressure up to 50 MPa on macroscopic
spray characteristics of a multi-hole gasoline direct injection injector
fueled with ethanol. Proc. Inst. Mech. Eng.,
Part D.

[12] Li X., Li D., Dimitriou P., Ajmal T., Aitouche A., Mobasheri R., Rybdylova O., Pei Y., Peng Z. (2023). Comparative investigation
on macroscopic and microscopic characteristics of impingement spray
of gasoline and ethanol from a GDI injector under injection pressure
up to 50 MPa. Energy Reports.

[13] Poling, B. ; Prausnitz, J. ; O’Connell, J. The Properties of Gases and Liquids, 5th ed.; McGraw-Hill: 2001.

[14] Kiran, E. ; Debenedetti, P. G. ; Peters, C. J. Supercritical Fluids: fundamentals and applications; Springer Science & Business Media: 2012; Vol. 366.

[15] Roy S., Askari O. (2020). A New Detailed Ethanol
Kinetic Mechanism at Engine-Relevant
Conditions. Energy Fuels.

[16] Zhang Q., Xia J., He Z., Wang J., Liu R., Zheng L., Qian Y., Ju D., Lu X. (2021). Experimental study
on spray characteristics of six-component diesel surrogate fuel under
sub/trans/supercritical conditions with different injection pressures. Energy.

[17] Harman-Thomas J. M., Hughes K. J., Pourkashanian M. (2022). The development
of a chemical kinetic
mechanism for combustion in supercritical carbon dioxide. Energy.

[18] Mardani A., Barani E. (2018). Numerical investigation of supercritical
combustion
of H2-O2. Energy Fuels.

[19] Liang W., Li W., Law C. K. (2019). Laminar
flame propagation in supercritical hydrogen/air
and methane/air mixtures. Proceedings of the
Combustion Institute.

[20] Plácido P. V. R., Alviso D., Gonçalves dos Santos R. (2024). A reduced
kinetic model for the oxidation of supercritical ethanol/gasoline
surrogate blends. J. Braz. Soc. Mech. Sci. Eng..

[21] Kogekar G., Karakaya C., Liskovich G. J., Oehlschlaeger M. A., DeCaluwe S. C., Kee R. J. (2018). Impact of non-ideal
behavior on ignition
delay and chemical kinetics in high-pressure shock tube reactors. Combust. Flame.

[22] Wang H., Ra Y., Jia M., Reitz R. D. (2014). Development of a reduced n-dodecane-PAH
mechanism and its application for n-dodecane soot predictions. Fuel.

[23] Yaws, C. L. Thermophysical Properties of Chemicals and Hydrocarbons; William Andrew: 2014.

[24] Marinov N.
M. (1999). A detailed
chemical kinetic model for high temperature ethanol oxidation. International Journal of Chemical Kinetics.

[25] Cancino L., Fikri M., Oliveira A., Schulz C. (2010). Measurement and chemical
kinetics modeling of shock-induced ignition of ethanol- air mixtures. Energy Fuels.

[26] Lee C., Vranckx S., Heufer K. A., Khomik S. V., Uygun Y., Olivier H., Fernandez R. X. (2012). On the chemical kinetics of ethanol
oxidation: shock tube, rapid compression machine and detailed modeling
study. Zeitschrift für Physikalische
Chemie.

[27] Konnov A., Meuwissen R., De Goey L. (2011). The temperature dependence of the
laminar burning velocity of ethanol flames. Proceedings of the Combustion Institute.

[28] Metcalfe W. K., Burke S. M., Ahmed S. S., Curran H. J. (2013). A hierarchical and
comparative kinetic modeling study of C1- C2 hydrocarbon and oxygenated
fuels. International Journal of Chemical Kinetics.

[29] Cai L., Pitsch H. (2015). Optimized chemical
mechanism for combustion of gasoline
surrogate fuels. Combust. Flame.

[30] Marques C. S., da Silva J. R. (2021). Reduced reaction
mechanisms for ethanol under ultra-lean
conditions in internal combustion engines. ACS
omega.

[31] Shi X., Qian W., Wang Q., Luo H., Kang Y., Ni J. (2021). Effect of
water content of hydrous ethanol on chemical kinetic characteristics
based on the new developed reduced ethanol-toluene reference fuels
mechanism. Fuel.

[32] Jin Y., Ma Z., Wang X., Liu F., Li X., Chu X. (2023). Experimental
and Kinetic Study of the Effect of Nitrogen Dioxide on Ethanol Autoignition. ACS omega.

[33] Li J., Kazakov A., Dryer F. L. (2004). Experimental and numerical studies
of ethanol decomposition reactions. J. Phys.
Chem. A.

[34] Li, J. ; Kazakov, A. ; Chaos, M. ; Dryer, F. L. Chemical kinetics of ethanol oxidation. In 5th US combustion meeting. 2007; pp 25–28.

[35] Zyada A., Samimi-Abianeh O. (2019). Ethanol kinetic
model development and validation at
wide ranges of mixture temperatures, pressures, and equivalence ratios. Energy Fuels.

[36] Zhang Y., El-Merhubi H., Lefort B., Le Moyne L., Curran H. J., Keromnes A. (2018). Probing the
low-temperature chemistry of ethanol via
the addition of dimethyl ether. Combust. Flame.

[37] Rabitz H., Kramer M., Dacol D. (1983). Sensitivity
analysis in chemical
kinetics. Annu. Rev. Phys. Chem..

[38] Pepiot-Desjardins P., Pitsch H. (2008). An efficient error-propagation-based
reduction method
for large chemical kinetic mechanisms. Combust.
Flame.

[39] Mestas P., Clayton P., Niemeyer K. (2019). pyMARS: automatically reducing chemical
kinetic models in Python. J. Open Source Software.

[40] Mittal G., Burke S. M., Davies V. A., Parajuli B., Metcalfe W. K., Curran H. J. (2014). Autoignition of ethanol in a rapid compression machine. Combust. Flame.

[41] Song Y., Zheng Z., Xiao J. (2019). Development
and validation of a reduced
chemical kinetic mechanism for supercritical gasoline of GDI engine. Fuel.

[42] Hinton N., Stone R., Cracknell R. (2018). Laminar burning velocity measurements
in constant volume vessels-Reconciliation of flame front imaging and
pressure rise methods. Fuel.

[43] Dong S., Wagnon S. W., Maffei L. P., Kukkadapu G., Nobili A., Mao Q., Pelucchi M., Cai L., Zhang K., Raju M. (2022). A new detailed kinetic
model for surrogate fuels: C3MechV3.3. Appl.
Energy Combust. Sci..

[44] Span, R. Multiparameter equations of state: an accurate source of thermodynamic property data; Springer Science & Business Media: 2013.

[45] Green, D. W. ; Southard, M. Z. Perry’s chemical engineers’ handbook; McGraw-Hill Education: 2019.

[46] Goodwin, D. G. ; Moffat, H. K. ; Schoegl, I. ; Speth, R. L. ; Weber, B. W. Cantera: An Object-oriented Software Toolkit for Chemical Kinetics, Thermodynamics, and Transport Processes, 2023, Version 3.0.0. https://www.cantera.org.

[47] Hibbert, D. B. Compendium of terminology in analytical chemistry; Royal Society of Chemistry: 2023.

[48] Heller S. R., McNaught A., Pletnev I., Stein S., Tchekhovskoi D. (2015). InChI, the
IUPAC international chemical identifier. J.
Cheminf..

[49] Joback, K. G. A unified approach to physical property estimation using multivariate statistical techniques. Ph.D. thesis, Massachusetts Institute of Technology: 1984.

[50] Owczarek I., Blazej K. (2003). Recommended critical
temperatures. Part I. Aliphatic
hydrocarbons. J. Phys. Chem. Ref. Data.

[51] Plácido P. V.
R., Alviso D., Gonçalves dos Santos R. (2024). A Reduced
Kinetics Model for the Oxidation of Transcritical/Supercritical Gasoline
Surrogate/Ethanol Mixtures Using Real Gas State Equations. Combust. Sci. Technol..

[52] Tang W., Brezinsky K. (2006). Chemical kinetic simulations behind reflected shock
waves. International journal of chemical kinetics.

[53] Tereshchuk P., Da Silva J. L. (2012). Ethanol and water
adsorption on close-packed 3d, 4d,
and 5d transition-metal surfaces: A density functional theory investigation
with van der Waals correction. J. Phys. Chem.
C.

[54] Heufer, K. ; Uygun, Y. ; Olivier, H. ; Vranckx, S. ; Lee, C. ; Fernandes, R. Experimental study of the high-pressure ignition of alcohol based biofuels. In Proc. European Combust Meeting; 2011.

[55] Gülder Ö. L. (1982). Laminar burning velocities of methanol, ethanol and isooctane-air
mixtures. Symposium (international) on combustion..

[56] Egolfopoulos F. N., Du D., Law C. K. (1992). A study
on ethanol oxidation kinetics in laminar premixed
flames, flow reactors, and shock tubes. Symposium
(international) on combustion..

[57] Bradley D., Lawes M., Mansour M. (2009). Explosion bomb measurements
of ethanol-air
laminar gaseous flame characteristics at pressures up to 1.4 MPa. Combust. Flame.

[58] Liao S., Jiang D., Huang Z., Zeng K., Cheng Q. (2007). Determination
of the laminar burning velocities for mixtures of ethanol and air
at elevated temperatures. Applied Thermal Engineering.

[59] Van
Lipzig J., Nilsson E., De Goey L., Konnov A. (2011). Laminar burning
velocities of n-heptane, iso-octane, ethanol and their binary and
tertiary mixtures. Fuel.

[60] Dirrenberger P., Glaude P.-A., Bounaceur R., Le Gall H., Da Cruz A. P., Konnov A., Battin-Leclerc F. (2014). Laminar burning
velocity of gasolines
with addition of ethanol. Fuel.

[61] Monteiro, J. O. D. Laminar flame speed of fuel mixtures applied to spark ignition internal combustion engines. PhD thesis, Universidade Federal de Santa Catarina: Florianópolis, SC, 2015, Available at https://repositorio.ufsc.br/xmlui/handle/123456789/169283.

[62] Alviso D., Garcia A., Mendieta M., Gonçalves dos Santos R., Darabiha N. (2022). Experimental and kinetic
modeling studies of laminar
flame speed of n-butanol/ethanol blends. J.
Braz. Soc. Mech. Sci. Eng..

[63] Zheng L., Figueroa-Labastida M., Nygaard Z., Ferris A. M., Hanson R. K. (2024). Laminar
flame speed measurements of ethanol, iso-octane, and their binary
blends at temperatures up to 1020 K behind reflected shock waves. Fuel.

[64] Rau F., Hartl S., Voss S., Still M., Hasse C., Trimis D. (2015). Laminar burning
velocity measurements using the Heat
Flux method and numerical predictions of iso-octane/ethanol blends
for different preheat temperatures. Fuel.

