# Supplementary material for: Nonideal State Equations to Evaluate the Laminar Flame Speed and Ignition Delay Times at Subcritical, Transcritical, and Supercritical Conditions of Ethanol
Source: ACS Omega. 2025 May 30;10(22):22445–58. doi: 10.1021/acsomega.4c09415 (PMC12163791; doi:10.1021/acsomega.4c09415)
Supplement: Supplementary file 1 [file ao4c09415_si_001.pdf]

Supporting Information : ACS Omega

Paulo Vitor Ribeiro Plácido*[a], Henrique Beneduzzi Mantovani[a], Dario Alviso[b,c], Rogério Gonçalves dos Santos[a]

[a]School of Mechanical Engineering, University of Campinas (UNICAMP), Rua Mendeleyev, 200 - CEP 13083-860, Cidade Universitária "Zeferino Vaz", Barão Geraldo, Campinas/SP, Brazil
[b]Laboratorio de Mecánica y Energía, Facultad de Ingeniería, Universidad Nacional de Asunción, Campus Universitario, CP 2160, San Lorenzo, Paraguay
[c]Universidad de Buenos Aires/CONICET, Laboratorio de Fluidodinámica, Facultad de Ingeniería, Paseo Colón 850, CP 1063, Buenos Aires, Argentina

---

Article title: Non-ideal state equations to evaluate the laminar flame speed and ignition delay times at subcritical, transcritical and supercritical conditions of ethanol

1. Thermodynamic properties: the molar Helmholtz free energy

As available in Cantera [1] and implemented by Kogekar *et al.* [2], the thermodynamics properties are obtained considering the multi-component mixture expression of the P-R and R–K cubic EoS (Eq 1, and Eq. 2) integrated into the definition of Helmholtz free energy (a) of any species f (Eq. S1). It is related to a reference state °, which is chosen to be an ideal gas at the given chemical composition and temperature (T) [K] at a reference pressure p° in the ideal gas state. Thus, the resulting Helmholtz energy departure function of a multi-component mixture can be re-written as in Equation S2 while using R-K EoS or written as in Equation S3 when using P-R EoS :

$$p \equiv \left(\frac{\partial a}{\partial v}\right)_{n_f,T} \tag{S1}$$

$$n_T(a - a^\circ) = -RTn_T \ln\left(\frac{p^\circ(V - n_T b^\star_{mix})}{n_T RT}\right) - \frac{n_T a^\star_{mix}}{b^\star_{mix}\sqrt{T}} \ln\left(\frac{V + n_T b^\star_{mix}}{V}\right) \tag{S2}$$

$$n_T(a - a^\circ) = -\frac{n_T \cdot a^\star \alpha}{2\sqrt{2b^\star_{mix}}} \ln\left(\frac{V + (1+\sqrt{2})n_T \cdot b^\star_{mix}}{V + (1-\sqrt{2})n_T \cdot b^\star_{mix}}\right) - n_T \cdot RT \ln\left(\frac{p^\circ(V - n_T \cdot b^\star_{mix})}{n_T RT}\right) \tag{S3}$$

Where the new terms, such as v represent the molar volume [m$^3 mol^{-1}$], n_T [mol] corresponds to the total number of moles of the multi-component mixture, converting molar volume v to total volume $V = n_T v$, and the molar Helmholtz energy [J mol^{-1}] is expressed by a.

In Equation S2 and S3, the parameter $a^\circ(T)_f$ corresponds to (u°_f - Ts°_f) and signifies the reference state Helmholtz energy for any species f or a mixture. This energy is determined by the internal energy u°_f and entropy s°_f based on the reference state of the ideal gas law adopted (p°, T, X_f). Thus, reference properties can be derived from the temperature-dependent properties $u^\ominus_f$ and $s^\ominus_f$, known as standard-state ($\ominus$), which are calculated using NASA polynomials [3, 4] (i.e., u°_f(T, X$_f$) = $u^\ominus_f$(T), and s°_f(T, X$_f$) = $s^\ominus_f$(T)- Rln(X$_f$)).

From the Helmholtz energy departure function in Eq. S2 or in Eq. S3, it is possible to calculate other significant thermodynamic properties, such as the specific enthalpy (h), internal energy (u), entropy (s), Gibb's free energy (g),

*Corresponding author.
Email address: vitorvrp@gmail.com; p228244@dac.unicamp.br (Paulo Vitor Ribeiro Plácido)

through the following correlations [5].

$$(h - h^{\circ}) = (a - a^{\circ}) + T(s - s^{\circ}) + RT(Z - 1) \tag{S4}$$

$$(u - u^{\circ}) = (a - a^{\circ}) + T(s - s^{\circ}) \tag{S5}$$

$$(s - s^{\circ}) = -\left(\frac{\partial(a - a^{\circ})}{\partial T}\right)_{v} = \int_{\infty}^{v}\left[\left(\frac{\partial p}{\partial T}\right)_{v} - \frac{R}{v}\right]dv + R\ln\frac{v}{v_{\circ}} \tag{S6}$$

$$(g - g^{\circ}) = (a - a^{\circ}) + RT(Z - 1) \tag{S7}$$

Furthermore, the fugacity coefficient ($f^{\star}$) can be expressed similarly as:

$$\ln\frac{f^{\star}}{p} = \frac{a - a^{\circ}}{RT} + \ln\frac{v}{v^{\circ}} + (Z - 1) - \ln Z \tag{S8}$$

$$\ln\frac{f^{\star}}{p} = -\frac{1}{RT}\int_{\infty}^{v}\left(p - \frac{RT}{v}\right)dv + (Z - 1) - \ln Z \tag{S9}$$

where Z is the compressibility factor.

1.1. Chemical kinetics of real gases: effects on mass action kinetics

Another important property is the departure function for the chemical potential (μ_f) of any species f from the reference state (μ_f°) at a constant volume, temperature, and constant moles number for all species j except f. This function is expressed as:

$$(\mu_f - \mu_f^{\circ}) = \left(\frac{\partial(n_T a - n_T a^{\circ})}{\partial n_f}\right)_{T,V,n_{j\neq f}} \tag{S10}$$

This property is part of the definition of the species relative activity coefficient (α_f) for real gas, $\alpha_f = \exp\left(\frac{\mu_f - \mu_f^{\circ}}{RT}\right)$, which measures the effective concentration of a species in a mixture. The species activity concentration of f is expressed as $[C_{ac,f}] = \alpha_f[X_f^{\circ}]$, where $[X_f^{\circ}]$ is the thermodynamic state reference molar concentration of species f ($\text{mol}_f \cdot \text{m}^{-3}$ for an ideal gas $p^{\circ}X_f R^{-1}T^{-1}$).

Furthermore, an activity coefficient (γ_f) adjusts the concentration in ideal conditions. Similarly, partial pressures can be scaled for gases using a fugacity coefficient ($f^{\star}$) to account for deviations from ideal behavior, representing a departure from ideal conditions. Therefore, the activity coefficient γ_f of species f is related to the activity concentration $[C_{ac,f}]$ of species f as $[C_{ac,f}] = \gamma_j[X_f]$.

The interesting idea here is that the real gas behavior influences the mass kinetics through the activity concentration $[C_{ac,f}]$ of any species f for a reversible i^{th} reaction under elevated pressure. According to many authors [6–9], for a reversible ith reaction, the rate of progress variable (ROP, $\dot{q}_i$) under elevated pressure can be expressed using the mass-action kinetics law:

$$\dot{q}_i = \left(k_{f,i}\prod_{f=1}^{N}[C_{ac,f}]^{\nu'_{f,i}} - k_{r,i}\prod_{f=1}^{N}[C_{ac,f}]^{\nu''_{f,i}}\right) \tag{S11}$$

Here, $k_{r,i}$ and $k_{f,i}$are the reverse and forward rate constants, and $\nu''_{f,i}$ and $\nu'_{f,i}$ are the reverse and forward stoichiometric coefficients for species f in the ith reversible reaction. The activity concentration of species f is defined as $[C_{ac,f}]$.

Finally, the species activity coefficients for real gas are obtained by combining the multi-component mixture total Helmholtz energy departure function obtained using a real gas state equation (Eq. S2 using R-K EoS or Eq. S3 using P-R EoS), the definition of the activity coefficients (α_f and γ_f), the departure function of chemical potential, Eq. (S10), and by replacing the term of the reference state total Helmholtz energy $n_T a^{\circ} = \sum_j n_f(a_f^{\ominus} + RT\ln X_f)$. After all these steps, the Readlich-Kwong and Peng-Robinson species activity coefficient expression can be obtained, respectively, as in Eq. (S12) converting total volume $V = n_T v$, exactly as in Kogekar *et al.* [2] for R-K EoS, and can be obtained as Eq. (S13) for P-R EoS.

$$RT\ln(\gamma_f) = RT\ln\left(\frac{v}{v-b^{\star}_{mix}}\right) + RT\left(\frac{b^{\star}_f}{v-b^{\star}_{mix}}\right) + \frac{a^{\star}_{mix}b^{\star}_f - 2\left(b_{mix}\sum_j a^{\star}_{jf}X_j\right)}{b^2_{mix}\sqrt{T}}\ln\left(\frac{v+b^{\star}_{mix}}{v}\right) - \frac{a^{\star}_{mix}}{b^{\star}_{mix}\sqrt{T}}\left(\frac{b^{\star}_f}{v+b^{\star}_{mix}},\right) \quad \text{(S12)}$$

$$RT\ln(\gamma_f) = -RT\ln\left(\frac{pV}{n_T RT}\right) + RT\ln\left(\frac{V}{V-n_T b^{\star}_{mix}}\right) + \frac{b_f n_T RT}{(V-n_T b^{\star}_{mix})} - \left[\frac{2\sum_j n_j(a^{\star}\alpha)_{f,j}}{2\sqrt{2n_T b^{\star}_{mix}}} - \frac{(n_T^2 a^{\star}\alpha)_{mix}b_f}{2\sqrt{2n_T^2 b^{\star 2}_{mix}}}\right]\ln\left(\frac{V+(1+\sqrt{2})n_T b^{\star}_{mix}}{V+(1-\sqrt{2})n_T b^{\star}_{mix}}\right) - \frac{(n_T^2 a^{\star}\alpha)_{mix}b_f V}{\left(V+(1-\sqrt{2})n_T b^{\star}_{mix}\right)\left(V+(1+\sqrt{2})n_T b^{\star}_{mix}\right)(n_T b_{mix})} \quad \text{(S13)}$$

1.2. 1D Non-ideal Flame equations

The flame is modeled in Cantera as stabilized in an axisymmetric stagnation flow [1]. It computes the solution along the stagnation streamline (r=0) using a similarity solution to reduce the three-dimensional (3D) governing equations (continuity, radial momentum, energy, and species continuity) to a single dimension (1D), as detailed in Kee *et al.* [10]. Additionally, the boundary conditions used here are for an adiabatic, freely propagating flame.

Concerning the steady-state, quasi-one-dimensional reacting flows (1D) non-ideal flame equations, the main distinction is highlighted in the energy equation (S14) implemented in the Cantera version 3.0.0 [1]. The enthalpy (h) of the mixture depends on the temperature, pressure, and composition (h = h(T, P, Y_f)), and it is correlated with the Helmholtz energy departure function obtained using the Peng-Robinson (P-R) EoS or the Redlich-Kwong (R-K) EoS. Consequently, the specific heat (c_p) defined in terms of enthalpy is also changed from the ideal gas to P-R EoS and R-K EoS.

$$\rho u c_p \frac{\partial T}{\partial Z} = \frac{\partial}{\partial Z}\left(\lambda\frac{\partial T}{\partial Z}\right) - \sum_{f=1}^{F} c_{pf} j_{f,Z}\frac{\partial T}{\partial Z} - \sum_{f=1}^{F} h_f \dot{\omega}_f W_f \quad \text{(S14)}$$

where ρ is the density, u is the axial velocity, c_p is the heat capacity at constant pressure, c_{pf} is the specific heat capacity of species f, h_f is the enthalpy of species f, W_f is the molecular weight of species f, $\dot{\omega}_f$ is the molar production rate of species f, j_f is the diffusive mass flux of species f, T is the temperature, λ is the thermal conductivity.

2. Hydrous ethanol laminar flame speed (LFS)

Ethanol can be mixed with water in any proportion, and at the same time, water results as a product in ethanol burning. As the introduction mentions, hydrous ethanol can be utilized in HCCI engines [11], leading to higher thermal efficiency and reduced CO and HC exhaust emissions [12, 13]. Therefore, it is worthwhile to investigate the influence of water on ethanol's LFS at varying water levels for the ethanol-reduced M. The numerical results of the "Ethanol RM_2nd" model, depicting the LFS at 2 bar with 380 K and 450 K, are presented in Figure S1 for ethanol-water blends with 20% v/v and 40% v/v of water. Increasing the temperature raises LFS, while higher water content decreases it, consistent with the experimental findings of Hinton *et al.* [14]. The maximum LFS values are slightly above the stoichiometric point (ϕ = 1.1). The "Ethanol RM_2nd" model demonstrates excellent agreement when 20% of liquid volume of water forms the ethanol/water mixture, with an RMSE of about 1.5 to 2.3 cm/s. However, this RMSE increases to 2.5 - 4.00 cm/s when 40% of liquid volume of water makes up the mixture, as summarized in Table S1.

Additionally, as per Liang *et al.* [15], higher water content more significantly reduces the flame temperature for equivalence ratios above 1.0 compared to those below 1.0, showing a stronger influence of water on the temperature of the fame in rich mixture region, as presented in Figure S1. It occurs due to water's chemical and physical effects on ethanol combustion. According to Shi *et al.* [16], Bozorgmehr and Murray [17], higher water content physically reduces the combustible gas calorific value, increases the mixture's heat capacity, and absorbs some of the fuel's released heat, ultimately lowering the adiabatic flame temperature. Additionally, as reported by Hinton *et al.* [14], adding water dilutes the concentration of oxygen and ethanol, increasing the likelihood of collisions between water and ethanol molecules and decreasing the likelihood of collisions between oxygen and ethanol. On the other hand, chemical influence primarily occurs when combustion products comprise water molecules. Indeed, as per Shi *et al.* [16], the increase in water slows down the forward reaction rate of the combustion reaction and inhibits combustion.

Moreover, as anticipated from the prior results, when using the equations of state (R-K and P-R EoS) at 2 atm, no deviation from the ideal LFS is observed. Therefore, there is no justification for using real gas under these conditions.

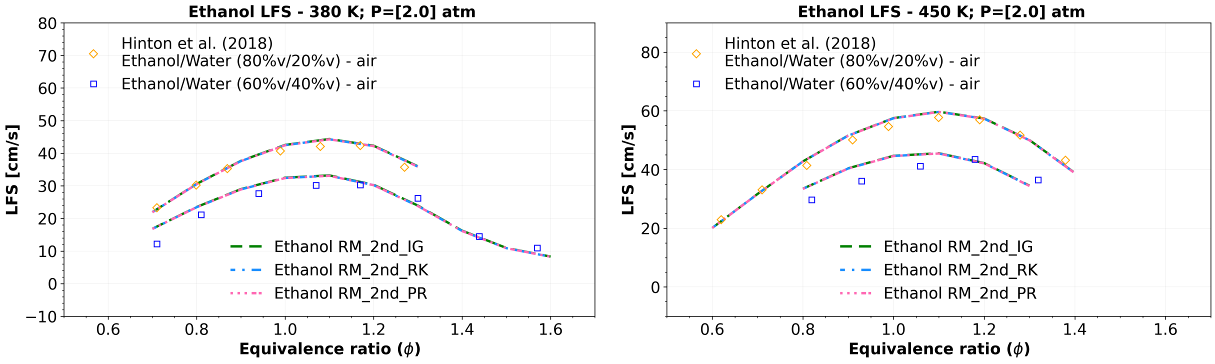

(a) (b)

Figure S1: Numerical LFS simulations of a mixture composition of Ethanol/Water, O_2, and N_2 comparing the "Ethanol RM_2nd" using ideal gas EoS (_IG) (▬ ▬), R-K EoS (_RK) (▬ · ▬), and P-R EoS (_PR) (· · · ▬) at a) 380 K, and b) 450 K, versus symbols representing experiments of LFS data at 2 atm from Hinton *et al.* [14].

Table S1: LFS RMSE of the "Ethanol RM_2nd" model against distinct Hydrous Ethanol LFS experimental data. In addition, the relative deviations (%) of IDT simulations were analyzed using distinct cubic equations of states (Redlich-Kwong and Peng-Robinson), compared to the IDT simulations from the ideal EoS at the same pressure conditions.

Fig	Experiments			Kinetics model					
	Authors	Pressure	Temp.	Compo. E/W [%V/%V]	Ideal gas [s]	Real gas (R-K) [s]	Real gas (P-R) [s]	Rel. D. (R-K) [%]	Rel. D. (P-R) [%]
				Hydrous Ethanol LFS					
Fig. 1(a)	[14]	2 atm	380 K	(80%E/20%W)	1.525	1.481	1.470	0.276%	0.337%
Fig. 1(a)	[14]	2 atm	380 K	(60%E/40%W)	2.531	2.487	2.480	0.552%	0.659%
Fig. 1(b)	[14]	2 atm	450 K	(80%E/20%W)	2.308	2.314	2.316	0.181%	0.214%
Fig. 1(b)	[14]	2 atm	450 K	(60%E/40%W)	4.091	4.022	4.009	0.342%	0.406%

Compo., composition of liquid volume (%) of ethanol and water;

Rel. D., Relative deviation about the LFS simulations adopting the ideal EoS;

3. Species comprised in the ethanol-reduced M. (Ethanol RM_2nd).

Table S2: species comprised in the ethanol-reduced M. (Ethanol RM_2nd).

Name	InChI*	Structure species	Formula
H	InChI=1S/H	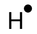	H
H2	InChI=1S/H2/h1H	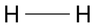	H2
O	InChI=1S/O	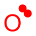	O
O2	InChI=1S/O2/c1-2	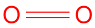	O2
OH	InChI=1S/HO/h1H	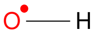	OH
H2O	InChI=1S/H2O/h1H2	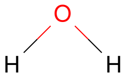	H2O
N	InChI=1S/N	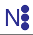	N
N2	InChI=1S/N2/c1-2	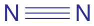	N2
HO2	InChI=1S/HO2/c1-2/h1H	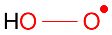	HO2
H2O2	InChI=1S/H2O2/c1-2/h1-2H	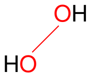	H2O2
AR	InChI=1S/Ar	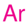	Ar
CO	InChI=1S/CO/c1-2	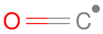	CO
CO2	InChI=1S/CO2/c2-1-3	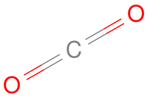	CO2
CH2O	InChI=1S/CH2O/c1-2/h1H2	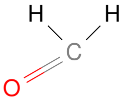	CH2O
HCO	InChI=1S/CHO/c1-2/h1H	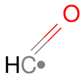	HCO

InChI*, InChI is a structure-based chemical identifier.

Name	InChl*	Structure species	Formula
HOCHO	InChI=1S/CH2O2/c2-1-3/h1H,(H,2,3)	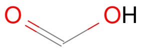	CH2O2
CH3OH	InChI=1S/CH4O/c1-2/h2H,1H3	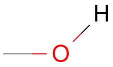	CH4O
CH3O2H	InChI=1S/CH4O2/c1-3-2/h2H,1H3	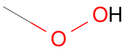	CH4O2
CH3O2	InChI=1S/CH3O2/c1-3-2/h1H3	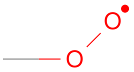	CH3O2
CH4	InChI=1S/CH4/h1H4	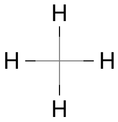	CH4
CH3	InChI=1S/CH3/h1H3	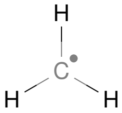	CH3
CH2	InChI=1S/CH2/h1H2	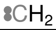	CH2
C	InChI=1S/C	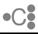	C
CH	InChI=1S/CH/H1h	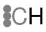	CH
C2H6	InChI=1S/C2H6/c1-2/h1-2H3	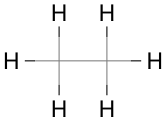	C2H6
C2H4	InChI=1S/C2H4/c1-2/h1-2H2	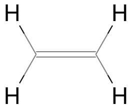	C2H4
C2H5	InChI=1S/C2H5/c1-2/h1H2,2H3		C2H5
C2H3	InChI=1S/C2H3/c1-2/h1H,2H2	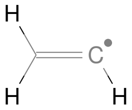	C2H3

InChI*, InChI is a structure-based chemical identifier.

Name	InChI*	Structure species	Formula
C2H2	InChI=1S/C2H2/c1-2/h1-2H	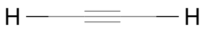	C2H2
C2H	InChI=1S/C2H/c1-2/h1H	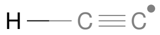	C2H
CH3CHO	InChI=1S/C2H4O/c1-2-3/h2H,1H3	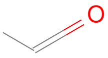	C2H4O
C2H3OH	InChI=1S/C2H4O/c1-2-3/h2-3H,1H2	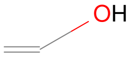	C2H4O
CH3CO	InChI=1S/C2H3O/c1-2-3/h1H3	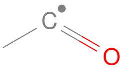	C2H3O
CH2CHO	InChI=1S/C2H3O/c1-2-3/h2H,1H2	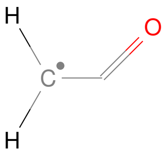	C2H3O
C2H3O1-2	InChI=1S/C2H3O/c1-2-3-1/h1H,2H2	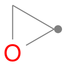	C2H3O
CH2CO	InChI=1S/C2H2O/c1-2-3/h1H2	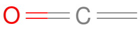	C2H2O
HCCO	InChI=1S/C2HO/c1-2-3/h1H	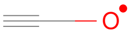	C2HO
CH3CO3H	InChI=1S/C2H4O3/c1-2(3)5-4/h4H,1H3	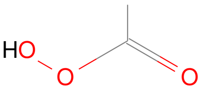	C2H4O3
CH3CO3	InChI=1S/C2H3O3/c1-2(3)5-4/h1H3	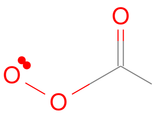	C2H3O3
CH3CO2	InChI=1S/C2H3O2/c1-2(3)4/h1H3	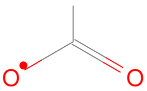	C2H3O2

InChI*, InChI is a structure-based chemical identifier.

Name	InChI*	Structure species	Formula
C2H5OH	InChI=1S/C2H6O/c1-2-3/h3H,2H2,1H3	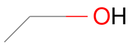	C2H6O
C2H5O	InChI=1S/C2H5O/c1-2-3/h2H2,1H3	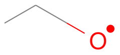	C2H5O
PC2H4OH	InChI=1S/C2H5O/c1-2-3/h3H,1-2H2	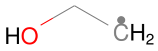	C2H5O
SC2H4OH	InChI=1S/C2H5O/c1-2-3/h2-3H,1H3	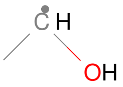	C2H5O
O2C2H4OH	InChI=1S/C2H5O3/c3-1-2-5-4/h3H,1-2H2	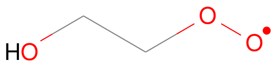	C2H5O3
C2H3CHO	InChI=1S/C3H4O/c1-2-3-4/h2-3H,1H2	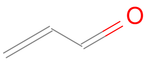	C3H4O
CH3CHCO	InChI=1S/C3H4O/c1-2-3-4/h2H,1H3	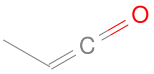	C3H4O
C3H6	InChI=1S/C3H6/c1-3-2/h3H,1H2,2H3	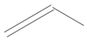	C3H6
C3H5-A	InChI=1S/C3H5/c1-3-2/h3H,1-2H2	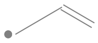	C3H5
C3H4-P	InChI=1S/C3H4/c1-3-2/h1H,2H3		C3H4
C3H4-A	InChI=1S/C3H4/c1-3-2/h1-2H2	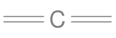	C3H4
C3H3	InChI=1S/C3H3/c1-3-2/h1H,2H2	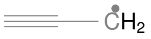	C3H3
C3H5OH	InChI=1S/C3H6O/c1-2-3-4/h2,4H,1,3H2	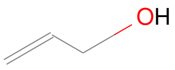	C3H6O

InChI*, InChI is a structure-based chemical identifier.

References

[1] Goodwin, D. G.; Moffat, H. K.; Schoegl, I.; Speth, R. L.; Weber, B. W. Cantera: An Object-oriented Software Toolkit for Chemical Kinetics, Thermodynamics, and Transport Processes. `https://www.cantera.org`, 2023; Version 3.0.0.

[2] Kogekar, G.; Karakaya, C.; Liskovich, G. J.; Oehlschlaeger, M. A.; DeCaluwe, S. C.; Kee, R. J. Impact of non-ideal behavior on ignition delay and chemical kinetics in high-pressure shock tube reactors. *Combustion and Flame* **2018**, *189*, 1–11.

[3] Kee, R. J.; Rupley, F. M.; Miller, J. A. *Chemkin-II: A Fortran chemical kinetics package for the analysis of gas-phase chemical kinetics*; 1989.

[4] McBride, B. J. *Coefficients for calculating thermodynamic and transport properties of individual species*; National Aeronautics and Space Administration, Office of Management . . . , 1993; Vol. 4513.

[5] Xiang, H. W. In *The Corresponding-States Principle and its Practice*; Xiang, H. W., Ed.; Elsevier: Amsterdam, 2005; pp 49–148.

[6] Eckert, C.; Boudart, M. On the use of fugacities in gas kinetics. *Chem. Eng. Sci* **1963**, *18*, 144–147.

[7] Érdi, P.; Tóth, J. *Mathematical models of chemical reactions: theory and applications of deterministic and stochastic models*; Manchester University Press, 1989.

[8] Froment, G. F.; Bischoff, K. B.; De Wilde, J. *Chemical reactor analysis and design*; Wiley New York, 1990; Vol. 2.

[9] Tang, W.; Brezinsky, K. Chemical kinetic simulations behind reflected shock waves. *International journal of chemical kinetics* **2006**, *38*, 75–97.

[10] Kee, R. J.; Coltrin, M. E.; Glarborg, P. *Chemically reacting flow: theory and practice*; John Wiley & Sons, 2005.

[11] Mack, J. H.; Aceves, S. M.; Dibble, R. W. Demonstrating direct use of wet ethanol in a homogeneous charge compression ignition (HCCI) engine. *Energy* **2009**, *34*, 782–787.

[12] Costa, R. C.; Sodré, J. R. Hydrous ethanol vs. gasoline-ethanol blend: Engine performance and emissions. *Fuel* **2010**, *89*, 287–293.

[13] Li, D.; Yu, X.; Sun, P.; Du, Y.; Xu, M.; Li, Y.; Wang, T.; Zhao, Z. Effects of water ratio in hydrous ethanol on the combustion and emissions of a hydrous ethanol/gasoline combined injection engine under different excess air ratios. *ACS omega* **2021**, *6*, 25749–25761.

[14] Hinton, N.; Stone, R.; Cracknell, R. Laminar burning velocity measurements in constant volume vessels–Reconciliation of flame front imaging and pressure rise methods. *Fuel* **2018**, *211*, 446–457.

[15] Liang, J.; Li, G.; Zhang, Z.; Xiong, Z.; Dong, F.; Yang, R. Experimental and numerical studies on laminar premixed flames of ethanol–water–air mixtures. *Energy & Fuels* **2014**, *28*, 4754–4761.

[16] Shi, X.; Qian, W.; Wang, Q.; Luo, H.; Kang, Y.; Ni, J. Effect of water content of hydrous ethanol on chemical kinetic characteristics based on the new developed reduced ethanol-toluene reference fuels mechanism. *Fuel* **2021**, *303*, 121201.

[17] Bozorgmehr, B.; Murray, B. T. Numerical simulation of evaporation of ethanol–water mixture droplets on isothermal and heated substrates. *ACS omega* **2021**, *6*, 12577–12590.
